# The *Drosophila* Nesprin-1 homolog MSP300 is required for muscle autophagy and proteostasis

**DOI:** 10.1242/jcs.262096

**Published:** 2024-06-10

**Authors:** Kevin van der Graaf, Saurabh Srivastav, Rajkishor Nishad, Michael Stern, James A. McNew

**Affiliations:** Department of BioSciences, Rice University, Houston, TX 77005, USA

**Keywords:** Aggregates, Autophagy, Endoplasmic reticulum, Polyubiquitin, ref(2)P, Atlastin

## Abstract

Nesprin proteins, which are components of the linker of nucleoskeleton and cytoskeleton (LINC) complex, are located within the nuclear envelope and play prominent roles in nuclear architecture. For example, LINC complex proteins interact with both chromatin and the cytoskeleton. Here, we report that the *Drosophila* Nesprin MSP300 has an additional function in autophagy within larval body wall muscles. RNAi-mediated *MSP300* knockdown in larval body wall muscles resulted in defects in the contractile apparatus, muscle degeneration and defective autophagy. In particular, *MSP300* knockdown caused accumulation of cytoplasmic aggregates that contained poly-ubiquitylated cargo, as well as the autophagy receptor ref(2)P (the fly homolog of p62 or SQSTM) and Atg8a. Furthermore, *MSP300* knockdown larvae expressing an mCherry–GFP-tagged *Atg8a* transgene exhibited aberrant persistence of the GFP signal within these aggregates, indicating failure of autophagosome maturation. These autophagy deficits were similar to those exhibited by loss of the endoplasmic reticulum (ER) fusion protein Atlastin (Atl), raising the possibility that Atl and MSP300 might function in the same pathway. In support of this possibility, we found that a GFP-tagged MSP300 protein trap exhibited extensive localization to the ER. Alteration of ER-directed MSP300 might abrogate important cytoskeletal contacts necessary for autophagosome completion.

## Teaser

**Summary:** The Nespin homolog MSP300 plays a role in basal autophagy in *Drosophila* muscle.

## INTRODUCTION

Members of the Nesprin (for ‘Nuclear envelope spectrin-repeat protein’) family are components of the linker of nucleoskeleton and cytoskeleton (LINC) complex (Hao and Starr, 2019; Kim et al., 2015; Luxton and Starr, 2014; McGillivary et al., 2023; Razafsky and Hodzic, 2009; Sosa et al., 2013) and play crucial roles in maintaining proper nuclear architecture and establishing nuclear–cytoskeletal connections (De Silva et al., 2023; Jahed et al., 2021). For example, the LINC complex is at least partly responsible for tethering the inner and outer nuclear membranes, mechanically coupling the nuclear membrane to the cytoskeleton and chromatin (Hao and Starr, 2019; Jahed et al., 2021) and properly positioning the nucleus within the cell (Burke, 2019; Lee and Burke, 2018; Volk, 2013; Wilhelmsen et al., 2006).

Multiple Nesprin domains mediate these various effects. For example, the Klarsicht/ANC-1/Syne-1 homology (KASH) domain (Jahed et al., 2021; Starr and Han, 2002), a C-terminal hydrophobic transmembrane segment that resides in the outer nuclear envelope, interacts with the SUN (for Sad1p–UNC-84) domain (Hao and Starr, 2019; Haque et al., 2010; Sosa et al., 2013, 2012; Zhou et al., 2012), which resides in the inner nuclear membrane. Nesprin cytoplasmic regions interact with a large variety of cytoskeletal elements, including F-actin [via a calponin homology (CH) domain in some isoforms], microtubules and microtubule motors (via spectrin repeats), and intermediate filaments (via plectin interaction) (De Silva et al., 2023; Luxton and Starr, 2014).

Nesprins function outside of the nuclear envelope as well. For example, brain-specific nesprin isoforms in rat generated by alternative splicing localize to the postsynaptic side of glutamatergic synapses and regulate glutamate receptor membrane trafficking (Cottrell et al., 2004), whereas nesprin isoforms lacking the KASH domain are found in synaptic regions in the mouse cerebellum (Razafsky and Hodzic, 2015). In *Drosophila*, the Nesprin ortholog MSP300 participates in localization of glutamate receptors as well as RNAs at the postsynaptic side larval neuromuscular junction. Larval muscle MSP300 forms ‘railroad tracks’ of immunoreactivity linking muscle nuclei and synapses, and encircles synaptic boutons (Morel et al., 2014; Packard et al., 2015). Finally, *C. elegans* deficient in the *Nesprin* ortholog *ANC-1* accumulate autophagic structures as well as undegraded cargo (Papandreou et al., 2023), indicating a role for Nesprins in autophagy progression.

Mutations in human nesprin orthologues are responsible for several genetic diseases. For example, nesprin mutations, primarily those affecting the KASH domain (Baumann et al., 2017; Puckelwartz et al., 2009) lead to muscle pathologies such as Emery Dreifuss muscular dystrophy (EDMD) or dilated cardiomyopathy (De Silva et al., 2023; Zhang et al., 2007; Zhou et al., 2017). Mutations in other LINC complex components can likewise lead to these diseases, suggesting that these pathologies arise specifically from LINC complex disruption (Janin et al., 2017; Janin and Gache, 2018). Nesprin mutations affecting neuron-specific splice variants can lead to the autosomal recessive disorder cerebellar ataxia 1 (ARCA1), which might reflect loss of nesprin isoforms lacking the KASH domain (Razafsky and Hodzic, 2015). However, the mechanisms linking nesprin loss with muscle or neuronal deficits remain incompletely understood.

The *Drosophila* system has been used to analyze effects of specific isoforms on specific molecular processes. Although the name MSP300 suggests a muscle-specific protein of 300 kDa, MSP300 is not muscle specific (Li et al., 2022; Zheng et al., 2020) and its 11 predicted transcripts generate products ranging in size from 47.3 kDa (497 amino acids, F-isoform) to 1.5 MDa (13,540 amino acids, G-isoform) (Elhanany-Tamir et al., 2012; Packard et al., 2015; Reuveny et al., 2018) (Fig. S1). The most prominent role for MSP300 is myonuclear positioning, consistent with its participation in the LINC complex (Elhanany-Tamir et al., 2012; Morel et al., 2014; Reuveny et al., 2018; van der Graaf et al., 2022; Wang et al., 2015). Recent work has evaluated the differential effects of specific MSP300 isoforms on muscle function. For example, eliminating the KASH or CH domains affects larval locomotion, whereas eliminating isoforms lacking the CH domain had no effect on larval locomotion (Morel et al., 2014; Rey et al., 2021). Mutations in the second Drosophila *Nesprin* homolog, called *Klarsicht* (*klar*, CG17046), (Fischer-Vize and Mosley, 1994; Mosley-Bishop et al., 1999; Patterson et al., 2004) likewise conferred locomotor and myonuclear positioning defects, and hence this protein has been proposed to act in a partially redundant manner with MSP300 (Elhanany-Tamir et al., 2012).

Here, we use the *Drosophila* system to show that MSP300 functions in the autophagy pathway. Severe RNAi-mediated knockdown of *MSP300* from muscle caused myonuclear clustering, muscle degeneration and pupal lethality. However, less severe knockdown led to muscle accumulation of protein aggregates containing polyubiquitin (polyUB) that were also labeled with ref(2)P (the fly homolog of p62, also known as SQSTM) and Atg8a, two pathway markers of autophagy (Dikic and Elazar, 2018; Menzies et al., 2015; Nakatogawa, 2020; Zhao et al., 2021; Zhao and Zhang, 2019). Removal of the KASH domain conferred similar accumulation of polyUB aggregates. *MSP300* knockdown also promoted persistence of the GFP signal from an mCherry (mCh)–GFP-tagged Atg8a protein, indicating failure of autophagosome maturation. Many of these phenotypes are also observed in larval muscle lacking the endoplasmic reticulum (ER) fusion protein Atlastin (Atl) (Xu et al., 2017; Srivastav et al., 2024) raising the possibility that Atl and MSP300 act in the same pathway. In support of this possibility, we found that endogenously expressed, fluorescently labeled MSP300 was localized throughout the entire ER network. Our work suggests that muscle autophagy failure might underlie pathologies conferred by loss of Nesprins.

## RESULTS

### Reduction in MSP300 levels results in a concentration-dependent increase in nuclear clustering

**Fig. 1.**
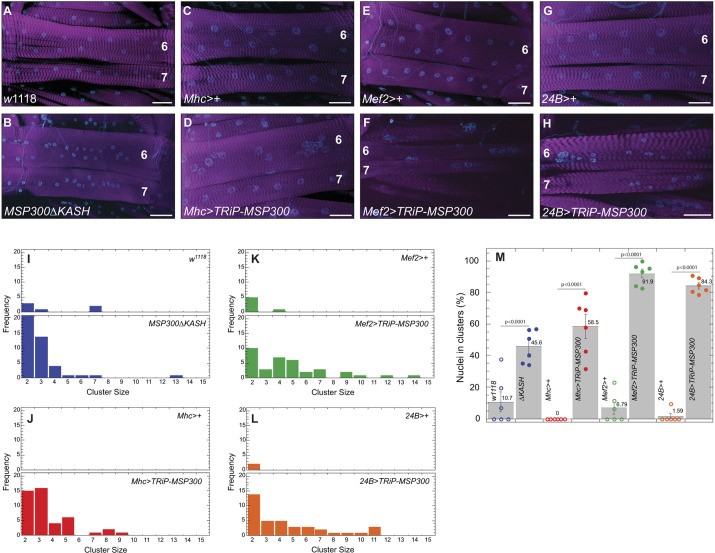
**Reduction in MSP300 levels results in a concentration-dependent increase in nuclear clustering.** (A–H) Phalloidin stain of filleted third-instar *Drosophila* larva of the indicated genotype. Nuclei are stained with DAPI. The body wall muscles quantified in this work include the ventral lateral longitudinal muscles (VL, labeled 6 and 7). Scale bars: 50 µm. (I–L) Frequency histogram of nuclear cluster size from ventral lateral longitudinal muscle 6 of the indicated genotype as per A–H. The total number of clusters is indicated for each genotype. *n*=six larvae from hemisegments A2–A4 (18 hemisegments total). (M) Percentage of total nuclei in clusters. Summary histogram of all nuclei in cluster of the indicated genotype. Individual animals are indicated as separate points (*n*=6) whereas control larvae are shown in open circle and experimental larvae are filled circles. Error bars are mean±s.e.m. are shown on the histogram, as well as the actual value of the mean. The *P*-values indicated are from a one-way ANOVA with a Tukey's post-hoc test.

One well-characterized phenotype of decreased MSP300 activity is a change in the location of muscle nuclei (Elhanany-Tamir et al., 2012; Morel et al., 2014; Reuveny et al., 2018; van der Graaf et al., 2022; Wang et al., 2015). For example, an RNAi construct that targets exon 23, a region common to all isoforms (Fig. S1), decreased levels of all *MSP300* transcripts and conferred muscle nuclear clustering (Rey et al., 2021). We, likewise, saw substantial nuclear clustering when we expressed an RNAi construct targeting exon 23, called *TRiP-MSP300*, in larval muscle (Fig. 1). Furthermore, we saw a strong correlation between the relative strength of the muscle Gal4 driver used and the degree of muscle nuclear clustering (Fig. 1A–H, quantified in Fig. 1I–M). Driving *TRiP-MSP300* expression with the relatively weak Myosin Heavy Chain (Mhc) Gal4 driver caused a somewhat mild change in nuclear clustering [58.5±7.4% (mean±s.e.m.) of nuclei in clusters of 2–3 nuclei, Fig. 1D,J,M], whereas the stronger Mef2 and 24B Gal4 drivers yielded larger nuclear clusters (Fig. 1F,H) with virtually all nuclei found in clusters (91.9±2.8% and 84.3±2.0%, respectively; Fig. 1M). Altered nuclear clustering was also observed in the *MSP300* truncation mutant Δ*KASH* (Fig. 1B) as was previously reported (Elhanany-Tamir et al., 2012; Morel et al., 2014), with 45.6±4.2% of nuclei found in clusters (Fig. 1M), predominantly in pairs (cluster size 2, Fig. 1I).

These changes in nuclear clustering were also mirrored by overall changes in muscle degeneration as measured by Alexa Fluor 647–phalloidin staining (Fig. S2). Whereas Mhc-driven *MSP300* knockdown resulted in very minor effects on overall muscle structure, Mef2- and 24B-driven knockdown resulted in dramatic alterations and degeneration of the muscle contractile apparatus (Fig. S2). Additionally, these latter genotypes resulted in 100% pupal lethality (Fig. S2J) as well as a significant (>90%) reduction in anterior spiracle eversion (Fig. S2K), a phenotype previously observed in mutants defective in larval muscle function (van der Graaf et al., 2021).

### Loss of MSP300 is accompanied by accumulation of large aggregates containing polyUB in the cytoplasm

**Fig. 2.**
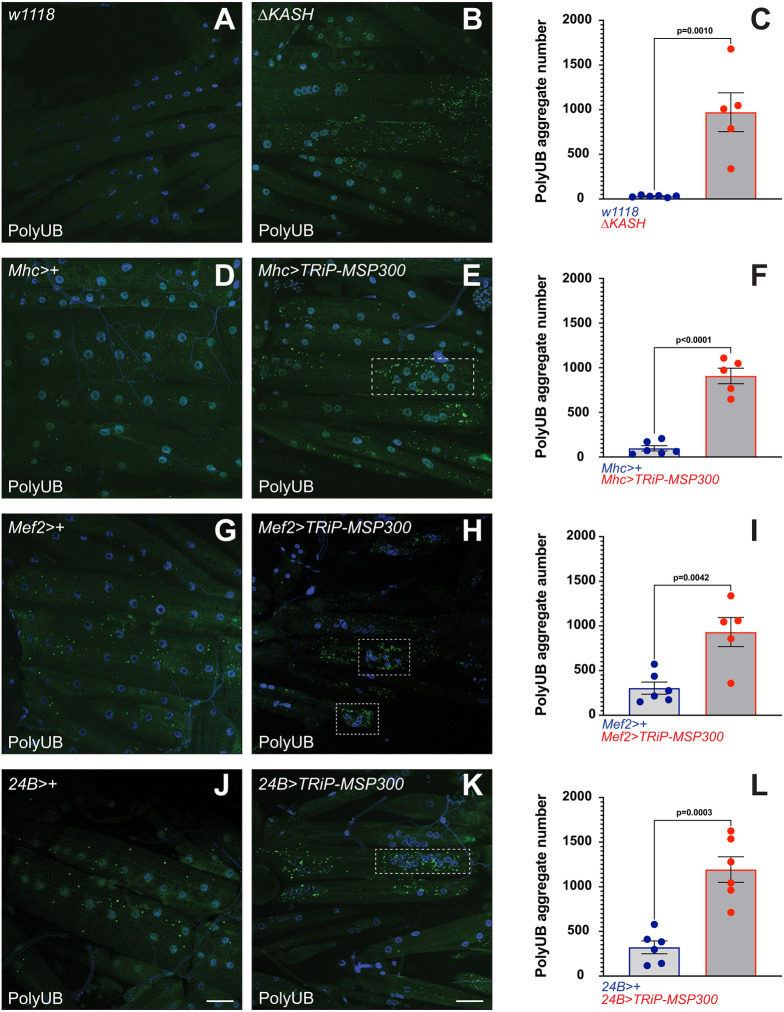
**Loss of MSP300 is accompanied by accumulation of large polyUB aggregates in cytoplasmic regions adjacent to the clustered nuclei.** Third-instar larvae were processed, fixed and stained with for polyUB (green) and DAPI (blue), then imaged by confocal microscopy. A single maximum intensity *z*-projection is shown. Scale bars: 50 µm. Quantification of polyUB aggregates from at least five individuals was performed using IMARIS (Bitplane). (A) Control (*w1118*), (B) *MSP300*Δ*KASH*, (D) *Mhc>+*, (E) *Mhc>TRiP-MSP300*, (G) *Mef2>+*, (H) *Mef2>TRiP-MSP300*, (J) *24B>+*, (K) *24B>Trip-MSP300*. (C,F,I,L) Histogram of total polyUB aggregates from the indicated genotypes. Individual animals are indicated as separate points (*n*=5 or 6). Mean±s.e.m. are shown on the histogram. The *P*-values indicated are from two-tailed unpaired Student's *t*-test analysis.

Our analysis of MSP300 function in autophagy began when we discovered that *attP40*, an insertion used for phiC31-dependent transgene integration (Groth et al., 2004; Thorpe and Smith, 1998), was an insertion mutation of *MSP300* (van der Graaf et al., 2022). During these studies, we found that muscle from *attP40-*bearing larvae contained large numbers of protein aggregates containing polyUB (Fig. S3). To determine whether polyUB aggregate accumulation occurred in other *MSP300* mutants as well, we examined aggregate accumulation in muscle from Δ*KASH* larvae and likewise found significant increases (Fig. 2A–C). In addition, *MSP300* knockdown with the *TRiP-MSP300* RNAi construct also caused significant aggregate accumulation (Fig. 2D–L). The extent of aggregate accumulation appeared to be largely independent of the degree of knockdown (Fig. 2D–L). Some of the larger aggregates tended to localize near the clustered nuclei (dashed boxes in Fig. 2E,H,K).

### The polyUB-containing aggregates are marked with the autophagy receptor ref(2)P and Atg8a

**Fig. 3.**
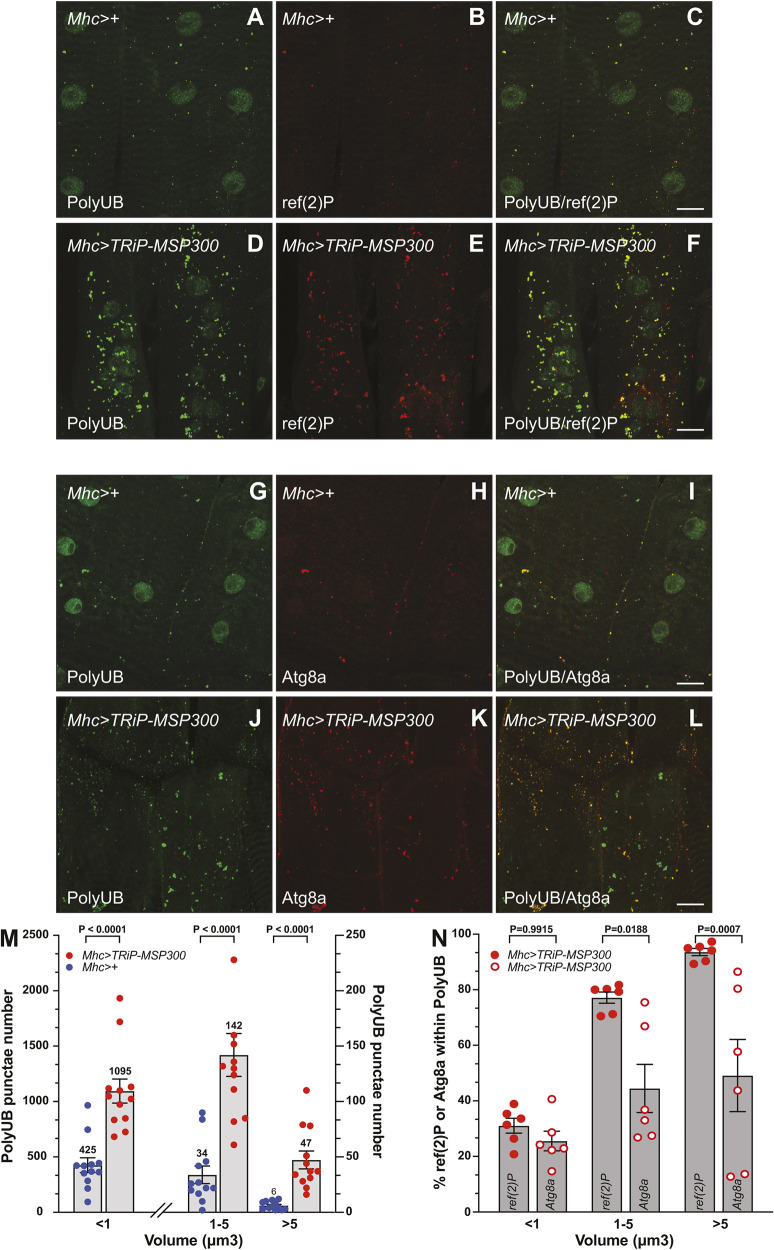
**Reduction in MSP300 causes polyUB accumulation marked with the autophagy intermediates ref(2)P and Atg8a.** (A–L) Muscle RNAi knockdown of *MSP300*. Confocal imaging (maximum intensity *z*-projections) of fixed third-instar larval musculature. Scale bars: 20 µm. (A–F) Antibodies against polyUB (green, A,D) and ref(2)P (red, B,M) in wild-type control larva (*Mhc>+*, A–C) and the *MSP300* knockdown mutant (*Mhc>Trip-MSP300*, D–F). C and F represent overlayed images of polyUB (green) and ref(2)P (red). (G–L) Antibodies against polyUB (green, G,J) and Atg8a (red, H,K) in wild-type control larva (*Mhc>+*, G–I) and the *MSP300* knockdown mutant (*Mhc>Trip-MSP300*, J–L). I and L represent overlayed images of polyUB (green) and Atg8a. (M) Size distribution of polyUB-positive surfaces (in cubic microns) within the volume examined in A, D, G and H. Imaging data was quantified using Imaris v9.7.2. Quantitative data are presented as mean±s.e.m. with actual value of the mean shown. Individual animals are indicated as separate points (*n*=12). Size data were binned to <1 µm^3^, 1–5 µm^3^ and >5 µm^3^. Note the left *y*-axis describes the histogram for <1 µm^3^ volume whereas the right *y*-axis scales the 1–5 µm^3^ and >5 µm^3^ histograms. The *P*-values indicated are from a two-tailed unpaired Student's *t*-test analysis. (N) Colocalization of ref(2)P or Atg8a with polyUB in *MSP300* knockdown larvae. The percentage of Imaris-generated surfaces of ref(2)P (filled red circles) or Atg8a (open red circles) that lies within surfaces generated by polyUB (similar to Manders M1 coefficient) are shown for size fractionated data (<1 µm^3^, 1–5 µm^3^ and >5 µm^3^). Quantitative data are presented as a mean±s.e.m. percentage. Individual animals are indicated as separate points (*n*=6). The *P*-values indicated are from a two-tailed unpaired Student's *t*-test analysis.

PolyUB-containing aggregate accumulation is often associated with a deficiency in autophagy (Pandey et al., 2007; Snyder et al., 2003; Yao, 2010). To determine whether these polyUB aggregates were likely autophagy intermediates, we colocalized polyUB with two autophagy intermediates: first, ref(2)P, the fly homolog of p62 (SQSTM), which is the primary polyUB receptor for non-selective autophagy in *Drosophila* (Lamark et al., 2017), and second, Atg8a, which is required for autophagosome formation and is responsible for binding to ref(2)P (Dikic and Elazar, 2018; Johansen and Lamark, 2020; Lorincz et al., 2017; Varga et al., 2022). We found that the polyUB aggregates generated by Mhc-Gal4-driven loss of MSP300 colocalized with both ref(2)P (Fig. 3D–F) and Atg8a (Fig. 3J–L), supporting the hypothesis that these aggregates are incompletely processed intermediates in autophagy.

We also examined the size of polyUB aggregates generated by MSP300 reduction by size-binning aggregates into three size populations. The majority of aggregates were relatively small (less than 1 µm^3^) in both control and MSP300 RNAi larvae; for these aggregates, MSP300 knockdown conferred only a 2.6-fold increase in number (Fig. 3M). However, larger aggregates were more strongly affected by MSP300 loss. In particular, for aggregates between 1 and 5 µm^3^, and greater than 5 µm^3^, MSP300 knockdown increased aggregate number 4.2-fold and 7.8-fold, respectively (Fig. 3M). For reference, a 1 µm^3^ sphere would have a diameter of ∼1.24 µm and a 5 µm^3^ sphere would have a diameter of ∼2.12 µm.

Interestingly, we observed a significant difference in the extent of Atg8a and ref(P)2 colocalizing with the larger polyUB aggregates relative to the smaller polyUB aggregates (Fig. 3N). Smaller polyUB aggregates (<1 µm^3^) were equally labeled with Atg8a and ref(2)P (Fig. 3N, filled red circles vs open red circles). Given that ref(2)P binds directly to ubiquitin, whereas Atg8a binds to ref(2)P, the equal labeling of polyUB for these aggregates suggests that Atg8a is present in sufficient quantities to occupy most or all ref(2)P-binding sites. In contrast, the larger polyUB aggregates (Fig. 3N, 1–5 µm^3^ and greater than 5 µm^3^ histograms, red circles versus open red circles) showed a loss of Atg8a binding relative to ref(2)P, indicating that Atg8a fails to occupy significant numbers of ref(2)P-binding sites on these larger aggregates.

### Atg8a overexpression, in the form of mCh–GFP–Atg8a, restores polyUB-Atg8a colocalization

The inability of Atg8a to occupy ref(2)P-binding sites within the larger UB aggregates (Fig. 3N) raised the possibility that MSP300 knockdown might decrease cytoplasmic Atg8a levels. To address this issue, we first determined whether Mhc-mediated knockdown of MSP300 reduced the overall amount of Atg8a proteins. Protein extract from wild-type (*Mhc>+*) and *Mhc>TRiP-MSP300* larval pelts were interrogated by western blotting with an antibody that recognizes Atg8a (Fig. S4). Although the average amount of Atg8a was reduced in five biological replicates, this reduction was not statistically significant, suggesting that lowering MSP300 levels did not substantially impact Atg8a protein levels.

**Fig. 4.**
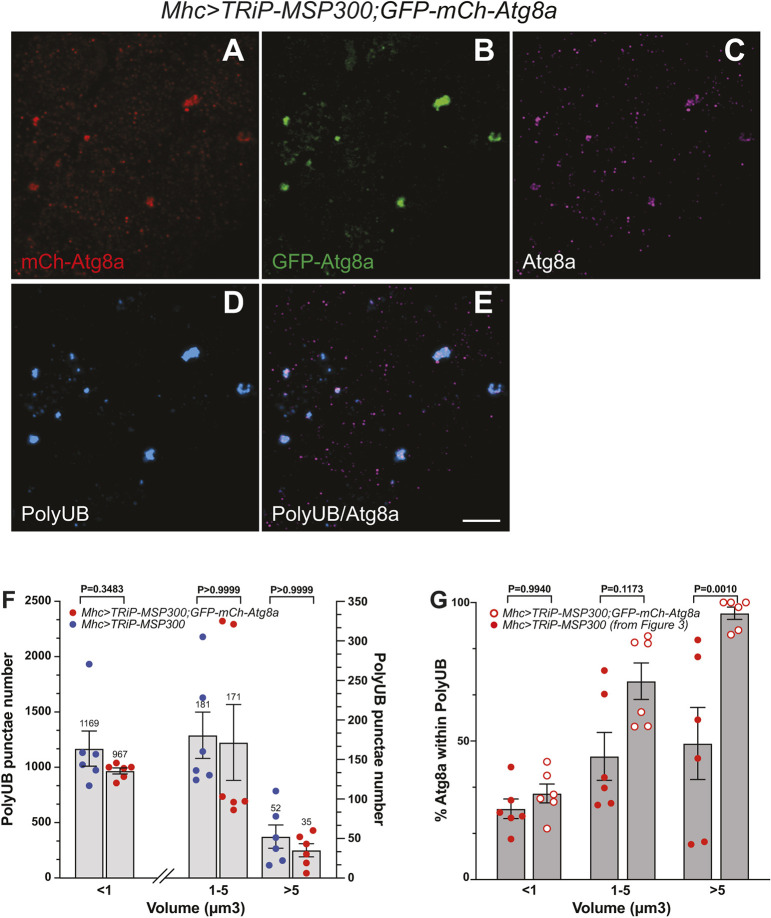
**Atg8a overexpression, in the form of mCh–GFP–Atg8a, restores polyUB–Atg8a colocalization without affecting polyUB aggregate accumulation.** (A–E) Confocal imaging (maximum intensity *z*-projections) of fixed third-instar of larval body wall muscle 6 expressing the autophagy marker *Atg8a*, tagged with both GFP and mCh in an MSP300 knockdown (*Mhc>TRiP-MSP300*) animal and immunolabelled with antibodies to polyUB and Atg8a. Scale bar: 5 µm. (A) mCherry (red), (B) GFP (green), (C) polyclonal antibodies against Atg8a (magenta) (D) monoclonal antibodies against polyubiquitin (cyan) and (E) merge of polyUB and Atg8a. (F) Size distribution of polyUB-positive surfaces (in cubic microns). Imaging data was quantified using Imaris v9.7.2. Quantitative data are presented as mean±s.e.m. with actual value of the mean shown. Individual animals are indicated as separate points (*n*=6). MSP300 knockdown larvae without Atg8a overexpression (*Mhc>TRiP-MSP300,* filled blue circles) and with Atg8a overexpression (*Mhc>TRiP-MSP300, GFP-mCh-Atg8a,* filled red circles) are shown. Size data were binned to <1 µm^3^, 1–5 µm^3^ and >5 µm^3^. Note the left *y*-axis describes the histograms for <1 µm^3^ volumes whereas the right *y*-axis scales the 1–5 µm^3^ and >5 µm^3^ histogram. The *P*-values indicated are from a two-tailed unpaired Student's *t*-test analysis. (G) Colocalization of Atg8a with polyUB in *MSP300* knockdown larvae with or without Atg8a overexpression. The percentage of Imaris-generated Atg8a surfaces that lies within surfaces generated by polyUB (similar to Manders M1 coefficient) are shown for size fractionated data (<1 µm^3^, 1–5 µm^3^ and >5 µm^3^) without (filled red circles) or with (open red circles) Atg8a overexpression. Quantitative data are presented as a mean±s.e.m. percentage. Individual animals are indicated as separate points (*n*=6). Note that the data for animals without Atg8a overexpression were taken from Fig. 3. The *P*-values indicated are from a two-tailed unpaired Student's *t*-test analysis.

Next, we tested the possibility that the incomplete labeling of larger aggregates with Atg8a was a result of Atg8a sequestration within the polyUB aggregates, thereby decreasing the available cytoplasmic Atg8a concentration. If so, we predicted that overexpressing *Atg8a* would significantly increase Atg8a labeling of even the largest polyUB aggregates. To test this prediction, we introduced an *Atg8a* transgene as a fluorescent chimera in which *GFP* and mCh were tagged in tandem to the N-terminus of Atg8a. We compared Atg8a localization in larvae expressing the *MSP300* RNAi transgene (*Mhc>TRiP-MSP300*) alone with larvae co-expressing the *MSP300* RNAi transgene and the Atg8a chimera (*Mhc>TRiP-MSP300*, *GFP-mCh-Atg8a*). We examined mCherry and GFP fluorescence as well as Atg8a and polyUB immunoreactivity in these larvae. Fig. 4 shows that large polyUB-positive aggregates (Fig. 4D) accumulated when MSP300 was reduced, as shown in Fig. 3 above. These structures also colocalized with both mCherry (Fig. 4A) and GFP (Fig. 4B). *Atg8a* overexpression failed to rescue polyUB aggregate number at any of the three puncta size tested (Fig. 4F). This result suggests that an inadequate supply of cytoplasmic Atg8a was unlikely to be the cause of aggregate accumulation in the MSP300 knockdown muscle. However, *Atg8a* overexpression significantly increased Atg8a labeling of the largest polyUB aggregates (Fig. 4C,G), suggesting that Atg8a sequestration within polyUB aggregates was responsible for the inability of Atg8a to fully occupy ref(2P) sites. Note that the data from control larvae (absence of *Atg8a* overexpression) was taken directly from Fig. 3.

### MSP300 reduction led to impaired autophagic flux and to accumulation of undegraded cargo

**Fig. 5.**
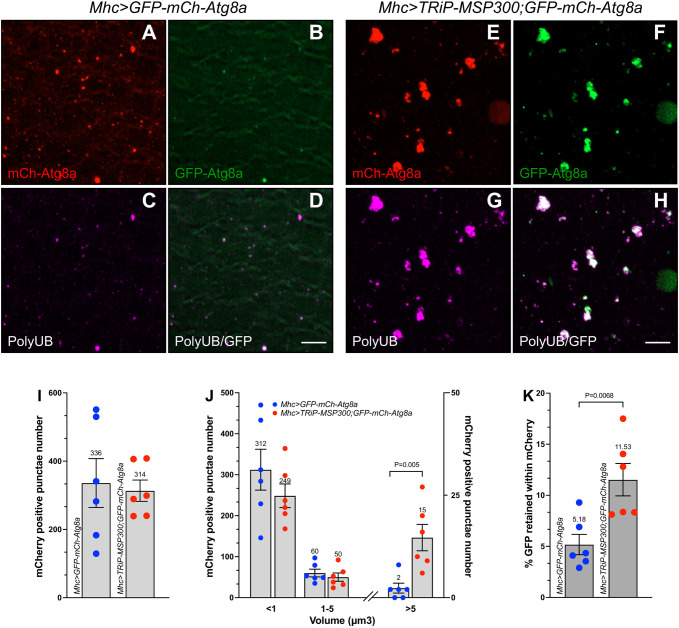
**MSP300 reduction leads to impaired autophagic flux and accumulation of undegraded cargo.** (A–H) Confocal imaging (maximum intensity *z*-projections) of fixed third-instar of larval body wall muscle 6. Scale bars: 5 µm. Wild-type animals expressing *Atg8a-mCherry-GFP* (*Mhc>GFP-mCh-Atg8a*) were imaged for mCherry (red, A), GFP (green, B) and stained with antibodies against polyUB (magenta, C). (D) Merged images of polyUB and GFP. MSP300 knockdown animals expressing *Atg8a-mCherry-GFP* (*Mhc>TRiP-MSP300, GFP-mCh-Atg8a*) were imaged for mCherry (red, E), GFP (green, F) and stained with antibodies against polyUB (magenta, G). (H) Merged images of polyUB and GFP. Data shown are representative of six different experiments (*n*=6). (I) Histogram of total mCh-positive surfaces from wild-type (*Mhc>GFP-mCh-Atg8a*, filled blue circles) and MSP300 knockdown animals (*Mhc>TRiP-MSP300, GFP-mCh-Atg8a,* filled red circles). Individual animals are indicated as separate points (*n*=6). Mean±s.e.m. are shown on the histogram, with actual value of the mean shown. The *P*-values indicated are from a two-tailed unpaired Student's *t*-test analysis. (J) Size distribution of mCh-positive surfaces (in cubic microns) from wild-type (*Mhc>GFP-mCh-Atg8a*, filled blue circles) and MSP300 knockdown animals (*Mhc>TRiP-MSP300, GFP-mCh-Atg8a,* filled red circles). Mean±s.e.m. are shown on the histogram with actual value of the mean shown. Individual animals are indicated as separate points (*n*=6). Size data were binned to <1 µm^3^, 1–5 µm^3^ and >5 µm^3^. Note the left *y*-axis describes the histograms for <1 µm^3^ and 1–5 µm^3^ volumes whereas the right *y*-axis scales the >5 µm^3^ histogram. The *P*-values indicated are from a two-tailed unpaired Student's *t*-test analysis. (K) Quantitation of GFP and mCherry colocalization. The percentage of Imaris-generated surfaces of GFP that lies within surfaced generated by mCherry for wild-type (*Mhc>GFP-mCh-Atg8a*, filled blue circles) and MSP300 knockdown animals (*Mhc>TRiP-MSP300, GFP-mCh-Atg8a,* filled red circles). Mean percentage±s.e.m. are shown on the histogram with actual value of the mean shown. Individual animals are indicated as separate points (*n*=6). The *P*-value indicated are from a two-tailed unpaired Student's *t*-test analysis.

We (Srivastav et al., 2024) and others (Nagy et al., 2015; Nezis et al., 2010) have previously used the GFP-mCh-Atg8a chimera to measure autophagic flux in *Drosophila*. *Drosophila* larval muscle expressing the Atg8a chimera alone (*Mhc>GFP-mCh-Atg8a*) or in combination with the *TRiP-MSP300* RNAi transgene were imaged for mCherry and GFP fluorescence as well as polyUB immunoreactivity. Autophagosomes incorporating the Atg8a chimera display both the mCherry and GFP signals; however, following fusion of autophagosomes with lysosomes, mCherry signal is retained whereas GFP signal is lost due to the acidic and/or degradative environment (Nagy et al., 2015). Wild-type animals (*Mhc>GFP-mCh-Atg8a)* showed a small number of mCh-positive puncta (Fig. 5A) containing very little GFP signal (Fig. 5B). This pattern is indicative of normal autophagic flux, in which autophagosomes are quickly converted into autolysosomes. In contrast, MSP300 knockdown larvae (*Mhc>TRiP-MSP300, GFP-mCh-Atg8a*) exhibited puncta containing polyUB (Fig. 5G) and GFP (Fig. 5F) in addition to mCh (Fig. 5E). Although overall, the number of mCh puncta was not different between wild-type and mutant animals (Fig. 5I), puncta size fractionation revealed that MSP300 knockdown significantly increased the number of particles greater than 5 µm^3^ (Fig. 5J). These observations confirm that large particles containing autophagic material preferentially accumulate in MSP300 knockdown larvae.

We noticed that MSP300 knockdown significantly increased the number of polyUB-containing but not mCh-containing puncta within the 1–5 µm^3^ population (Fig. 3M versus Fig. 5J). We suggest two explanations for this discrepancy. First, many polyUB puncta in MSP300 knockdown muscle do not contain Atg8a, even when overexpressed (∼70%, see Fig. 4G), and second, as described above, most mCh-containing puncta in control muscle are likely found in autolysosomes, which are not expected to contain polyUB. Most importantly, MSP300 knockdown animals but not controls accumulate a significant amount of GFP within mCh-positive structures (11.5% versus 5.2%, Fig. 5K) demonstrating impaired autophagic flux.

### MSP300 reduction increases Foxo activity

**Fig. 6.**
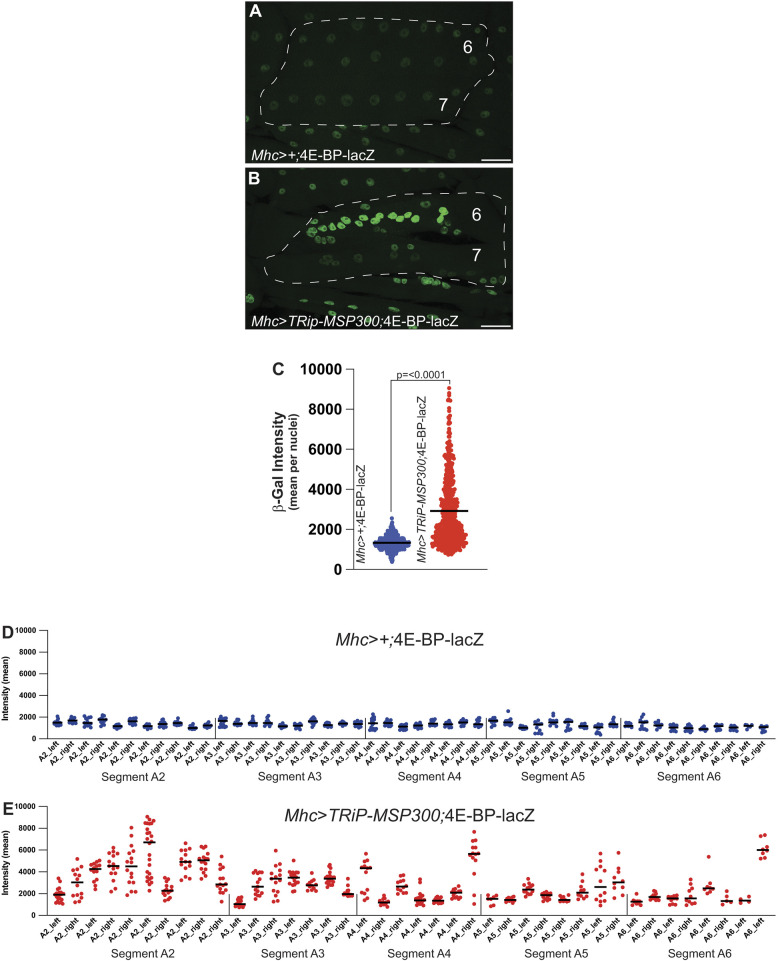
**MSP300 reduction increases Foxo activity.** Third-instar larvae were processed, fixed and stained with β-galactosidase antibodies, then imagined by confocal microscopy. (A,B) The Foxo-dependent transcriptional reporter *4E-BP-lacZ* expression was identified by antibody staining with β-galactosidase in wild-type (A, (*Mhc>+;*4E-BP-lacZ) and MSP300 knockdown animals (B, *Mhc>TRiP-MSP300;*4E-BP-lacZ). A single maximum intensity *z*-projection is shown. Scale bar: 50 µm. (C–E) Nuclear β-galactosidase was quantified. (C) Total β-galactosidase staining intensity for each nuclei is represented as mean intensity for wild-type (*n*=611 nuclei, filled blue circles) and MSP300 knockdown (*n*=529 nuclei, filled red circles). The *P*-value indicated is from a two-tailed unpaired Student's *t*-test analysis. (D) Nuclear β-galactosidase staining intensity for wild-type animals are segregated by abdominal segments, beginning at A2 through A6. Each graph represents β-galactosidase intensity in each hemisegment (left and right) from 5 to 6 animals, with the mean marked. (E) Nuclear β-galactosidase staining intensity for MSP300 knockdown animals are segregated by abdominal segments, beginning at A2 through A6. Each graph represents β-galactosidase intensity in each hemisegment (left and right) from 3 to 6 animals, with the mean marked.

An autophagy impairment causing accumulation of polyUB aggregates and retention of GFP signal in a GFP–mCh-tagged Atg8a has been reported previously for larval muscle lacking the ER fusion protein Atlastin (Atl) (Srivastav et al., 2024). The phenotypic similarity between loss of Atl and MSP300 in autophagy raised the possibility that other phenotypes conferred by *atl* loss might also be observed in *MSP300* knockdown muscle. One such phenotype conferred by *atl* loss is activation of the stress-induced transcription factor Foxo (Stern and McNew, 2021; Xu et al., 2017). To test the possibility that MSP300 knockdown would activate Foxo, we introduced the Foxo-dependent transcriptional reporter *4E-BP-lacZ*, in which a nuclear-localized β-galactosidase (β-gal) protein is transcribed and translated under the control of the *4E-BP* gene, into larvae expressing *TRiP-MSP300* under control of the Mhc Gal4 driver. Fig. 6 demonstrates that MSP300 knockdown conferred a significantly increased β-gal signal within larval muscle nuclei when imaged by immunocytochemistry (Fig. 6C). Somewhat surprisingly, we observed a heterogenous distribution of expression within nuclei of the same multinucleate syncytium as well as differences among muscle cells along the anterior–posterior axis (Fig. 6E). The second and third abdominal hemisegments appeared to show the greatest increase in 4E-BP expression, but more posterior segments sometimes showed increased expression as well. The mechanistic basis for this heterogeneity is unclear. Regardless, our results indicate that decreasing MSP300, like decreasing Atl, activates Foxo in larval muscles (Xu et al., 2017).

### Endogenous MSP300 occupies the entire ER

Eight of the 11 predicted MSP300 isoforms contain the nuclear-tethering KASH domain at the C-terminus (Fig. S1) and therefore locate to the nuclear envelope. As predicted, MSP300 localization via immunocytochemistry in fixed tissue shows strong immunoreactivity at the nuclear envelope, but also at the Z-disks and in ‘railroad tracks’ linking muscle nuclei with the neuromuscular junction (Elhanany-Tamir et al., 2012; Morel et al., 2014; Packard et al., 2015; Reuveny et al., 2018; Wang et al., 2015). An additional report examined has examined an internal fluorescent protein chimera (MSP300-Venus) in fixed tissue and showed a similar nuclear envelope and Z-disk localization (Rey et al., 2021).

The nuclear envelope is a subdomain of the continuous ER network that permeates the entire cytoplasmic space as an anastomosing network of tubules and sheet (Obara et al., 2023). The KASH domain of MSP300 and the SUN domain of inner nuclear envelope proteins associate in the inner nuclear membrane space (effectively a specialized region of the ER lumen) to form the LINC complex. Although this association is likely to retain some of the MSP300 within the nuclear envelope, it is currently unclear whether additional subdomains of the ER are accessible to MSP300. The identified residence of MSP300 deep within the musculature defined as Z-disk localization suggest that other environments are possible. Additionally, the phenotypic similarity between loss of MSP300 and loss of the ER fusion Atl, raised the possibility that MSP300 might also occupy other regions of the ER.

**Fig. 7.**
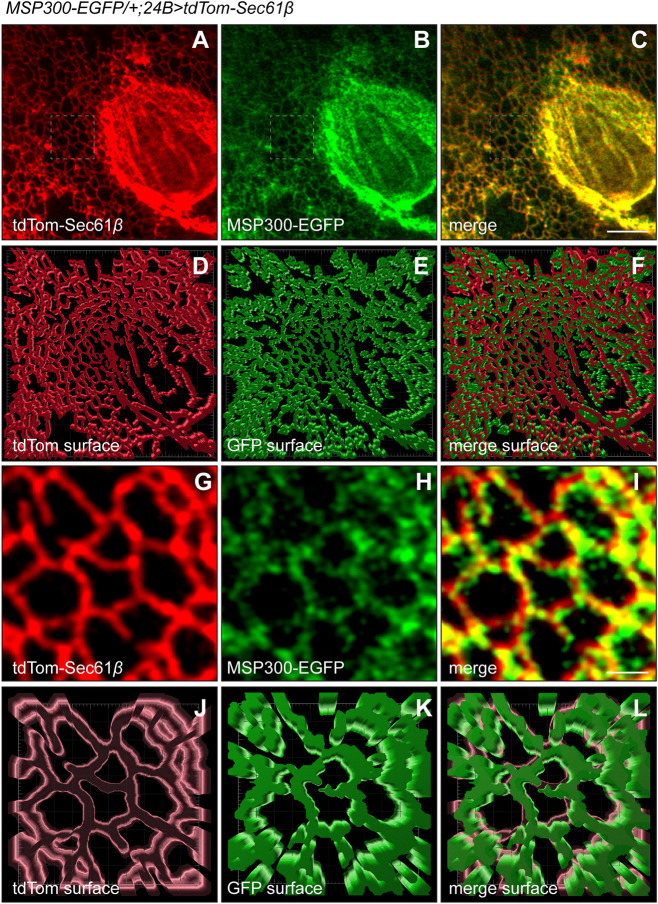
**MSP300 occupies the entire ER.** Live confocal imaging (maximum intensity *z*-projections) of larval body wall muscle 6 expressing the resident ER marker protein tdTomato–Sec61β (A, tdTom, red) and MSP300–EFGP (B, EGFP, green). (C) Merged image of tdTomato and EGFP. Note that these maximum intensity *z*-projections contain 0.6 µm of depth from the medial surface of the muscle. (D–F) Imaris surface modeling of the fluorescent images shown in A–C. Scale bars: 5 µm. (G–I) Enlarged (5×5 µm) image of the region indicated by the boxed area of A–C (tdTom, red), B (EGFP, green), and C (merge of TdTom and EGFP). (J–L) Imaris surface modeling of the fluorescent images shown in G–I. Scale bar: 1 µm. Images are representative of between three and six biological replicates.

Because the stability and continuity of the ER is highly susceptible to perturbation by chemical fixation (Summerville et al., 2016), we chose to examine MSP300 localization in live tissue. To visualize MSP300, we utilized an EGFP protein trap (Nagarkar-Jaiswal et al., 2015) with an insertion located within the intron between exons 33 and 34 (Fig. S1), which we refer to as MSP300–EGFP. To visualize the ER, we expressed the defined ER membrane marker tdTomato (tdTom)–Sec61β (Summerville et al., 2016) in larval muscle, driven by 24B. We then examined several regions of muscle 6 (Fig. S5) in hemisegment A3. Much to our surprise, we observed that MSP300–EGFP was found in all ER structures examined in addition to the nuclear envelope. Fig. 7A–C shows an approximate 25 µm×25 µm×0.6 µm volume of medial cytoplasm adjacent to a single muscle nucleus (see Fig. S5 for a schematic of larval body wall muscles). These images reveal a precise colocalization between the Sec61β and MSP300 within the nuclear envelope and the peripheral ER tubule network, with >85% overlap. The primary data shown in Fig. 7A–C are presented as surface rendering models generated by Imaris (Bitplane) in Fig. 7D–F. Enlarged 5 µm×5 µm×0.6 µm volumes are also shown to illustrate the fine detail and precise colocalization with the ER tubules (Fig. 7G–L). It is currently unclear whether the pointilized nature of the MSP300-GFP fluorescence is truly reflective of discrete spots within the ER membrane or a consequence of low (endogenous) levels of expression relative to the overexpressed marker.

Additional ER structures deeper within the tissue also show precise colocalization between MSP300–EGFP and tdTom–Sec61β (Fig. S5). These structures include an intermediately deep region (between 3–5 µm in Z-depth) that we call the ‘pillar’ zone (Fig. S5A–C) as well as structures deeper within the acto-myosin contractile apparatus (7–9 µm in Z-depth, Fig. S5D–F). However, we do not see the pronounced Z-disk localization observed by others. This discrepancy could be a result from our use of live rather than fixed tissue; alternatively, the protein trap used in our study might not capture the MSP300 isoform present in the Z-disk.

## DISCUSSION

Nesprin family members are required for proper nuclear positioning, especially in large multinucleate syncytial cells such as the *Drosophila* larval body wall muscle. Here, we show that the *Drosophila* Nesprin ortholog MSP300 has an unexpected role in autophagy. We found that graded reduction of MSP300 from larval muscles resulted in a progressively severe defect in myonuclear positioning (Fig. 1), consistent with previous reports (Elhanany-Tamir et al., 2012; Morel et al., 2014; Reuveny et al., 2018; van der Graaf et al., 2022; Wang et al., 2015), as well as larval muscle degeneration (Fig. S1 and Rey et al., 2021). Additionally, we found that MSP300 loss caused accumulation of cytoplasmic aggregates decorated with polyubiquitin (Fig. 2), as well as the autophagy components ref2(2)P and Atg8a (Fig. 3), indicating that MSP300 loss prevents autophagosome progression through the autophagy pathway. The largest (>5 µm^3^) aggregates were fully labeled with ref2(2)P but not Atg8a, indicating that cytoplasmic Atg8a levels were limiting for autophagosome maturation (Fig. 3). Whereas *Atg8a* overexpression rescued this incomplete autophagosome labeling, *Atg8a* overexpression failed to restore normal autophagy progression to MSP300-depleted muscle, indicating that limited Atg8a levels were not causal for autophagy failure (Fig. 4). When we expressed *Atg8a* tagged with mCh and GFP in MSP300-defective muscle, we found persistence of the GFP signal; as GFP fluorescence in autophagosomes is lost following fusion with lysosomes, this GFP persistence suggests that MSP300 loss prevents proper autophagosome maturation (Fig. 5). MSP300 loss also activated the stress-dependent Foxo transcription factor (Fig. 6). The strong similarity between these phenotypes of MSP300 loss to phenotypes conferred by loss of the ER-fusion protein Atl raised the possibility that Atl and MSP300 control autophagy through a similar pathway. This possibility is supported by our observation that an EGFP-tagged MSP300 protein trap localizes to the entirety of the extended ER network (Fig. 7). Below we discuss the implications of our findings in deciphering mechanisms of autophagosome progression, as well as the mechanisms underlying Nesprin-dependent muscle degeneration.

### Nesprin involvement in microtubule function might underlie both autophagy and nuclear positioning

The accumulation of cytoplasmic polyUB aggregates in muscle lacking MSP300 activity was a somewhat unexpected result. We suggest that Nesprins, in general, and MSP300 in particular, regulate both nuclear positioning and autophagy by providing a crucial adapter function between organelles and the cytoskeleton. Nesprins, including MSP300, interact directly or indirectly with all cytoskeletal tracks, including microtubules, actin filaments and intermediate filaments, as well motor proteins such as kinesins (De Silva et al., 2023; Jahed et al., 2021). In fact, myonuclear positioning in larval body wall muscles by MSP300 requires interaction with the *Drosophila* Spectraplakin Short stop (Shot), a cytoskeletal crosslinking protein, as well as the plus-end microtubule binding protein EB1 (Wang et al., 2015), (Akhmanova and Steinmetz, 2015; Applewhite et al., 2010; Nehlig et al., 2017; Zhao et al., 2022). MSP300 has also been implicated directly in the formation of cytoplasmic tracts involved in RNA localization in muscle (Packard et al., 2015). Microtubule motors participate in organelle positioning and movement (Bonifacino and Neefjes, 2017; Cabukusta and Neefjes, 2018; Oyarzun et al., 2019; Pu et al., 2016; Redpath and Ananthanarayanan, 2023) and play key roles in autophagy (Bejarano et al., 2018; Mackeh et al., 2013), which requires appropriate location and mobility of the participating organelles such as the ER, autophagosomes amphisomes and lysosomes (Bonifacino and Neefjes, 2017; Cabukusta and Neefjes, 2018; Dikic and Elazar, 2018; Fu and Holzbaur, 2014; Nakatogawa, 2020). For these reasons, we suggest that alteration in organelle movement caused by MSP300 loss might result in autophagy deficits and hence the abnormal accumulation of undegraded intermediates that we have observed.

### MSP300 localization within ER

Residence of MSP300 in the outer nuclear envelope is dependent on the presence of the C-terminal KASH transmembrane domain and is tethered to the inner membrane via interactions with SUN proteins (Gurusaran and Davies, 2021; Sosa et al., 2012) to form the LINC complex. We found that EGFP–MSP300 localized throughout the entire ER network. This observation suggests that it is possible that the entire LINC complex, not only MSP300, is also located within the peripheral ER tubular network potentially influencing the architecture of the ER tubule. In fact, the diameter of an ER tubule is similar to the distance between the inner and outer nuclear membranes (Cain and Starr, 2015; Georgiades et al., 2017). Alternatively, ER-localized MSP300 might represent one or more of the three predicted isoforms that lack the KASH domain and are therefore soluble. Recent work has shown that KASH-containing MSP300 isoforms have separate functions from KASH-less isoforms on larval locomotion, myonuclear positioning and localization (Rey et al., 2021). In this view, ER localization of these KASH-less isoforms would require interactions with ER-resident proteins. The presence of dozens of Spectrin repeats allow for ample opportunity to form higher-order structures through multiple protein-protein interactions.

### MSP300 function and disease

Mutations in genes encoding nesprins and other members of the LINC complex are associated with several human pathologies (De Silva et al., 2023; Horn, 2014; Storey and Fuller, 2022; van Heerden et al., 2023). Mutations in the nesprin *SYNE1* are most often found in spinocerebellar ataxia 8 (SCAR8) with these mutations distributed throughout the protein including the spectrin repeat region(s) and the CH domain, as well as the KASH domain (Storey and Fuller, 2022). Although many of these disorders affect tissues other than muscle, a connection between MSP300 function and autophagy likely expands the spectrum of disorders that can be attributed to deficits in the LINC complex. Additionally, MSP300 residence throughout the ER might directly influence ER structure and function. Several other ER-resident proteins, including the ER fusion protein Alt (McNew et al., 2013; Moss et al., 2011; Orso et al., 2009) also influence autophagy both directly and indirectly (Srivastav et al., 2024; Summerville et al., 2016; Xu et al., 2017). In fact, loss or reduction of *atl* function phenocopies some, but not all, MSP300 effects including accumulation of polyUB aggregates, reduction of autophagic flux and increases in Foxo activity (Srivastav et al., 2024). Finally, the results described in the work expand the interaction domain and functional consequence of MSP300 to include effects on autophagy.

## MATERIALS AND METHODS

### *Drosophila* stocks

All fly stocks were maintained on standard cornmeal/agar *Drosophila melanogaster* medium (69.1 g/l corn syrup, 9.6 g/l soy flour, 16.7 g/l yeast, 5.6 g/l agar, 70.4 g/l cornmeal, 4.6 ml/l propionic acid and 3.3 gm/l nipagin). The following *s*tocks were obtained from the Bloomington *Drosophila* stock center (BDSC, Bloomington, Indiana): *w^1118^* (3605), *4E-BP-lacZ* (9558), *UAS-Dcr-2* (24650), *UAS-pGFP-mCherry-Atg8a* (37749), 24B*-Gal4* (1767), TRiP-*MSP300* (32377) or (32848), Mef2-Gal4 (27390), MSP300-EGFP (59757), MSP300ΔKASH (26781). Flies bearing *Mhc-Gal4* were generously provided by Norbert Perrimon (HHMI Harvard Medical School, MA, USA). Flies carrying *attP2* and *attP40* lacking insertions were retrieved as white-eyed progeny from transgene insertions carried out at GenetiVision (Houston, TX, USA). All fly stocks were maintained on standard cornmeal/agar medium as previously described (van der Graaf et al., 2022). All experiments were performed on *Drosophila melanogaster* that had been reared and maintained at room temperature (RT; 22°C) with a 12-h-light–12-h-dark cycle unless otherwise indicated.

### Immunocytochemistry

Larvae were dissected in 1× phosphate-buffered saline (Gibco) with 0.3% Tween 20 (Thermo Fisher Scientific) (1× PBST) on sylgard plates (Xu et al., 2017). Dissected larvae were fixed with 4% paraformaldehyde for 10 min at RT followed by three washes with PBST for 10 min each. Larval samples were blocked by incubation with 1% bovine serum albumin (BSA; MP-Biomedicals) for 30 min. Samples were incubated overnight at 4°C with primary antibodies followed by three washes with PBST for 10 min each. Samples were then incubated overnight at 4°C with the secondary antibody followed by three washes with PBST. Samples were then mounted on slides using Vectashield containing DAPI (Vector laboratories). For experiments involving double immuno-staining with both mouse and rabbit primary antibodies, samples were first incubated overnight at 4°C with the rabbit primary antibody as described above, then incubated with Alexa Fluor 647-conjugated anti-rabbit-IgG secondary antibody (Abcam, ab150075) for 2 h at RT, followed by three washes of 10 min each with PBST. Samples were then incubated overnight at 4°C with the mouse primary antibody, followed by three washes of 10 min each with PBST. Samples were then incubated for 2 h at RT with Alexa Fluor 405 conjugated anti-mouse-IgG secondary antibody (Abcam, ab175658), followed by three washes of 10 min each with PBST.

Some larvae were dissected in HL3.1 (Feng et al., 2004) in a magnetic chamber, and fixed in 4% paraformaldehyde for 10 min, then washed in PBS with 0.3% Triton-X (PBS-T) and blocked for 30 min in PBS-T with 1% BSA.

The following primary antibodies were used in this work: mouse anti-polyubiquitin antibody (1:1000, EMD Millipore, ST1200-100UG); mouse anti-GLB1/β-galactosidase (1:500; Promega, Z378A), anti-GABARAP (Atg8a, 1:1000, Cell Signaling, E1J4E; rabbit mAb #13733) and rabbit anti-SQSTM1/p62 anti-peptide antibody (described previously, used at 1:100 dilution; Srivastav et al., 2024). The neuromuscular junctions were immunostained with rabbit anti-horseradish peroxidase (1:1000, Jackson ImmunoResearch Laboratories, AB_2340263) conjugated to Rhodamine Red. Finally, the primary rabbit anti-Atg8a anti-peptide antibody (used at 1:100 dilution) was generated by GenScript. A peptide (amino acids 1–15, MKFQYKEEHAFEKRR) derived from the extreme N-terminus of Atg8a-PA (CG32672) was appended with an N-terminal cysteine for coupling, was synthesized by GenScript, coupled to keyhole limpet haemocyanin (KLH), injected into 2 New Zealand rabbits, and purified by the manufacturer, similar to a previous report (Barth et al., 2011).

The following secondary antibodies were used in this work: goat anti-mouse-IgG coupled to FITC (1:1000; Abcam, ab6785), donkey anti-rabbit-IgG coupled to Alexa Fluor 647 (1:1000; Abcam, ab150075), and donkey anti-mouse-IgG coupled to Alexa Fluor 405 (1:1000; Abcam, ab175658). Alexa Fluor 647-conjugated phalloidin (1:200) was used to visualize F-actin.

### Nuclear clustering analysis

Third-instar larval body wall muscle 6 was chosen for all nuclear clustering analysis. Images were opened in ImageJ and nuclear clusters in muscle 6 were counted by a researcher who was aware of the experimental conditions. We defined a ‘cluster’ as two or more nuclei in which the distance between two nuclear borders was less than five microns (van der Graaf et al., 2022). We analyzed six larvae from hemisegments A2–A4 (18 hemisegments total) for each genotype. All clustering analysis was performed in Microsoft Excel as described previously (van der Graaf et al., 2022). Data were visualized and analyzed using GraphPad Prism v9.3.1 or Synergy Software KaleidaGraph v4.5.2.

### Pupal measurements

We measured the length in pupae for TRiP *MSP300* expression driven with each *Gal4* line plus their respective controls. Pupal length was determined from top to bottom, excluding the anterior and posterior spiracles. Pupal images were acquired with an Olympus trinocular microscope. ImageJ was used to measure lengths along the central axis beginning with the anterior end. Pupa lethality and spiracles were measured as previously described (van der Graaf et al., 2022).

### Fluorescence microscopy imaging

Fluorescence microscopy imaging of fixed larval tissue was performed on either a Zeiss LSM800 (Carl Zeiss, Jena, Germany) or a Nikon A1-Rsi inverted confocal microscope (Nikon, Tokyo, Japan). Zeiss LSM800 confocal microscope, apart from its regular confocal module is also equipped with an Airyscan-1 detector, which uses an GaAsP array detector consisting of 32 hexagonal micro lenses arranged in a circular disk. Images captured using an Airyscan GaAsP detector and subsequent deconvolution process resulted in a 2-fold resolution increase and 8-fold increase of signal-to-noise-ratio relative to conventional confocal microscopes (Wu and Hammer, 2021).

Airyscan super-resolution images were captured using a Plan-Apochromat 63×/NA DIC M27 water immersion objective in 1024×1024 frame size and pixel size at 0.21 µm. The excitation (ex) and emission (em) range are at the following wavelengths: ex 405 nm, em 410–480 nm (DAPI); ex 488 nm, em 493–570 nm (Alexa Fluor 488); ex 561, em 576–700 nm (Alexa Fluor 594) and ex 640 nm, em 640–700 nm (Alexa Fluor 647). Raw images captured were then processed under Airyscan Processing module available in Zen 2.6, Blue edition (Carl Zeiss Microscopy GmbH, Germany) with 2D SR processing option. The Airyscan filtering (Wiener filter associated with deconvolution) was set to Standard.

Imaging with the Nikon A1-Rsi confocal microscope was performed using Nikon NIS-Element software (Nikon, Tokyo, Japan) with either Plan Apochromat 20×/0.75 NA dry objective or 40×/1.15 NA water immersion objective. Image size was set at 1024×1024 resulted in pixel size 0.63 μm or 0.31 μm respectively while 2× averaging was applied in all imaging. Excitation laser wavelength and emission filters used were ex 405 nm, em 425–475 nm; ex 488 nm, em 500–550 nm; ex 561 nm, em 570–620 nm; and ex 640 nm, em 663–738 nm.

### Live sample preparation and imaging

Third-instar larvae were dissected on sylgard plates (Srivastav et al., 2024) in 1× Schneider's *Drosophila* medium (Gibco, 21720-024) to prepare larval fillets with body wall muscles exposed. All images were obtained from larval body wall muscle 6 from segment A2 and acquired with the Zeiss LSM800 Airyscan confocal microscope using Plan Apochromat 63×/1.2 NA water immersion objective with 1024×1024 image size and zoom factor was set at 3. Following acquisition, images were analyzed with Bit-Plane IMARIS v9.7.2.

### Image quantitative analysis

All fluorescence images acquired were quantified and analyzed using the Surface module of Imaris v9.7.2. (Bitplane, Zurich, Switzerland). In this module, the surface was created for the fluorophore signal of interest using background subtraction option followed by assigning a lower limit to voxel number. This lower limit was selected to minimize background noise and to avoid false positives in the surface creation. The surface was masked and the zero value was assigned to voxels outside of the surface. Surface volume, puncta number and mean intensity were obtained from the statistical data available for the surface created. The quantification of cytoplasmic polyUB required an initial step in which nuclei were masked based on DAPI staining to remove inconsistent nuclear polyUB signal. The statistics option was used to report the distribution of puncta based on volume.

Colocalization data were obtained with the ‘Object-Object Statistics’ option, whereby a surface for each fluorophore-bound protein of interest was created. Colocalization between two proteins was then determined with the ‘Overlapped Volume Ratio to Surfaces’ parameter under ‘Detailed Statistics’ using ‘Surface to Surface Overlapped’ MatLab-Imaris image analysis tools under Xtension module of the Imaris software. The ‘Surface to Surface Overlapped’ tool is based on Mander's colocalization method (Dunn et al., 2011).

### Measurement of Atg8a levels in larval pelts

Dissected larval pelts were collected and processed for protein extraction in lysis buffer [25 mM HEPES, 100 mM KCl, 2 mM EDTA, 1% Triton X-100 (Thermo Fisher Scientific; A16046AE), 50 mM sodium fluoride, 5 mM sodium orthovanadate, 1× Protease inhibitor cocktail (Roche, cat. no. 05892970001), 2 mM β-mercaptoethanol]. Pelts were homogenized in tubes containing lysis buffer (at 4°C) using a motorized micro pestle. Samples were then incubated on ice for 30 min and then centrifuged at 15,000 ***g*** for 30 min at 4°C. The supernatant from each tube was collected, and protein concentrations for each sample were determined using the Bradford method. For each sample, 60 µg of protein was applied to a 12% SDS-PAGE gel followed by electrophoresis at 90 V. Proteins were then transferred to a nitrocellulose membrane by blotting at 150 mA for 2 h at 4°C. Membranes were stained with Ponceau S to evaluate transfer efficiency and washed with 1× Tris-buffered saline with 1% Tween 20 (TBST; 150 mM NaCl, 50 mM Tris-HCl, pH 7.6, 1% Tween 20). Membranes were blocked by application of 5% powdered milk prepared in 1× TBST for 30 min. Membranes were then incubated overnight with primary antibodies at 4°C, followed by three washes in TBST for 10 min each. Membranes were then incubated with HRP-conjugated secondary antibodies for 2 h at RT with mild shaking, followed by three washes in TBST for 10 min each. Membranes were then incubated with ECL reagent and observed in a LAS 4000 gel imager (Fujifilm). Data obtained were quantified with the IMAGE Gauge v4.0 application (Fuji). The following primary antibodies were used: anti-GABARAP (Atg8a, 1:1000, Cell Signaling, E1J4E; rabbit mAb #13733), and mouse anti-Drosophila βTub/β-tubulin antibody (1:1000; Developmental Studies Hybridoma Bank, E7). The secondary antibody used was HRP-conjugated goat anti-mouse-IgG antibody (1:5000; Rockland 610-1319).

### Graphing and statistical analysis

All data analyzed in the present study were plotted and statistically analyzed using GraphPad Prism v9.3.1 or KaleidaGraph v5.0.6.

## Supplementary Material



10.1242/joces.262096_sup1 Supplementary information
