# Supplementary material for: The Drosophila Nesprin-1 homolog MSP300 is required for muscle autophagy and proteostasis
Source: J Cell Sci. 2024 Jun 10;137(11):jcs262096. doi: 10.1242/jcs.262096 (PMC11213522; doi:10.1242/jcs.262096)
Supplement: Supplementary information [file joces-137-262096-s1.pdf]

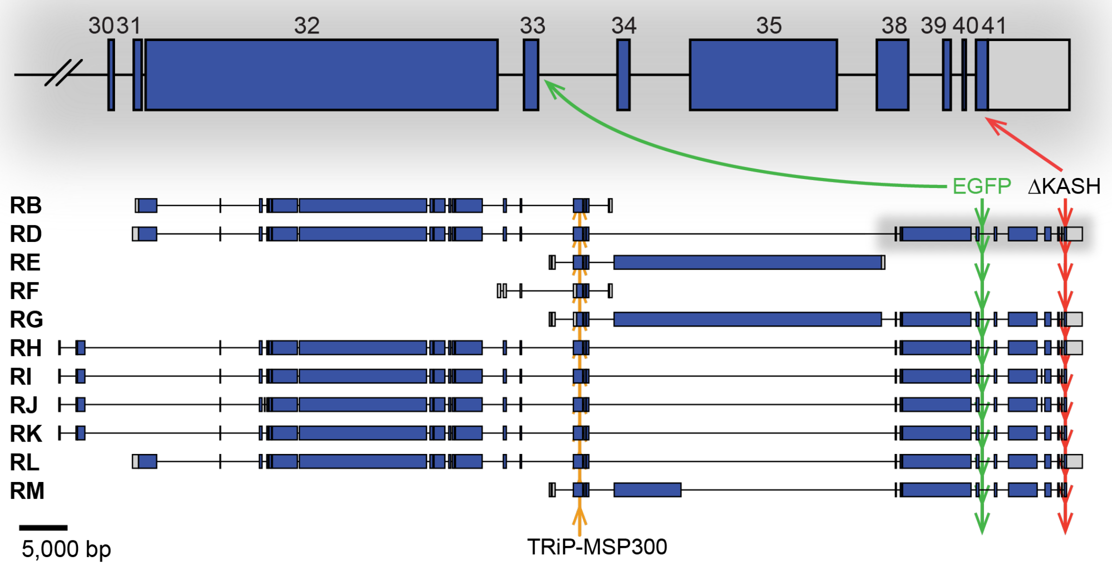

Fig. S1. MSP300 gene schematic and location of genomic reagents. The MSP300 gene (CG42768) encodes 11 isoforms by differential splicing of 42 exons. Eight isoforms (D,G,H,I,J,K,L and M) are predicted to include a C-terminal transmembrane span and a KASH domain expressed entirely from exon 41. A truncation mutant that eliminates the KASH domain is shown by a red arrow labelled ΔKASH. The location of a protein trap insertion is indicated by a green arrow between exons 33 and 34. This insertion codes for an in-frame fusion with a series of protein marker including EGFP [(GGS)4x-EGFP-FIAsH-StrepII-TEV-3xFLAG-(GGS)4x]. An enlarged diagram of the 5' region of the RD isoform is shown to more precisely detail the location of these insertions relative to the nearest exon. Finally, the location of a Transgenic RNAi Project (TRiP) insertion (32377 [y[1] sc[*] v[1] sev[21]; P{y[+t7.7] v[+t1.8]=TRiP. HMS00368}attP2] or (32848 [y[1] sc[*] v[1] sev[21]; P{y[+t7.7] v[+t1.8]=TRiP. HMS00632}attP2) expressing a double stranded RNA is indicated between by the orange arrows at exon 23. This TRiP insertion is predicted to interfere with all eleven isoforms of MSP300.

Journal of Cell Science • Supplementary information

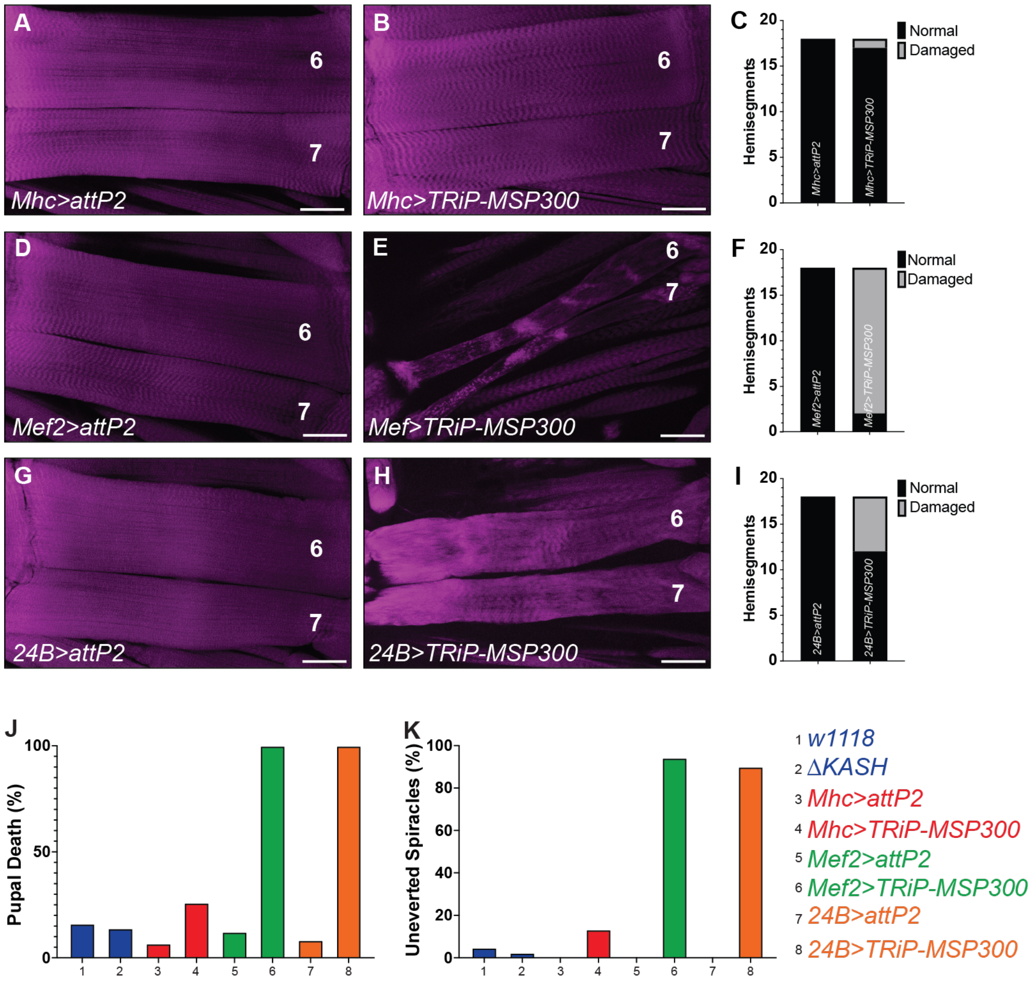

Fig. S2. Reduction in MSP300 levels results in a concentration -dependent increase in muscle degeneration. (**A, B, D, E, G, and H**) Phalloidin stain of filleted third instar Drosophila larva of the indicated genotype. Scale bar equals 50 µm. **(C, F, and I)** Stacked frequency histogram of larval hemisegments with normal (black) or damaged (gray) musculature determined phalloidin staining. Frequency of pupal death (**J**, percentage of total) and uneverted spiracles (**K**, percentage of total) of the indicated genoytypes. N=6 animals (18 hemisegments).

Journal of Cell Science • Supplementary information

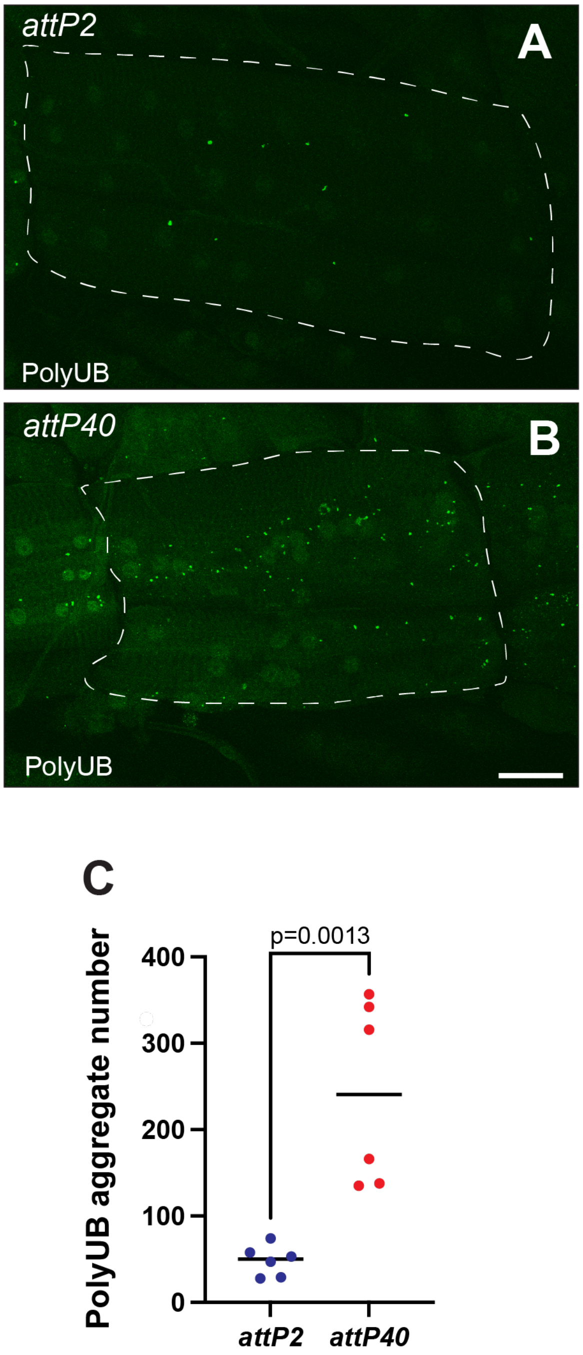

Fig. S3. ***att40*****, an insertional mutation of MSP300, shows mild cytoplasmic polyUB accumulation.** (**A and B**) Third instar larva were processed, fixed, and stained with polyUB antibodies, then imagined by confocal microscopy. A single maximum intensity z-projection of muscle 6 and 7 of the indicated genotype is shown. Scale bar equals 50 µm. **(C)** Quantitation of polyUB aggregates from 6 individuals by Imaris (Bitplane). Histogram of total polyUB aggregates from the indicated genotypes. Individual animals are indicated as separate points (n=6). Means are shown on the histogram. The p-values indicated are from a Student's t analysis.

Journal of Cell Science • Supplementary information

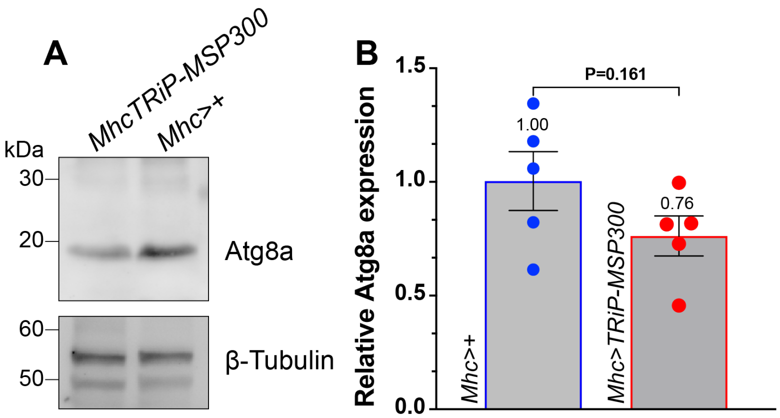

Fig. S4. Atg8a levels are slightly reduced in MSP300 knockdown. (**A**) Protein extracts larval pelts from the indicated genotypes were probed by western blotting. (B). Five independent biological replicates were quantified relative the b-Tubulin loading control and plotted as a histogram. Means ± SEM are plotted. The p-value from a Student's t analysis indicated the reduction in Atg8a levels in not statically significant.

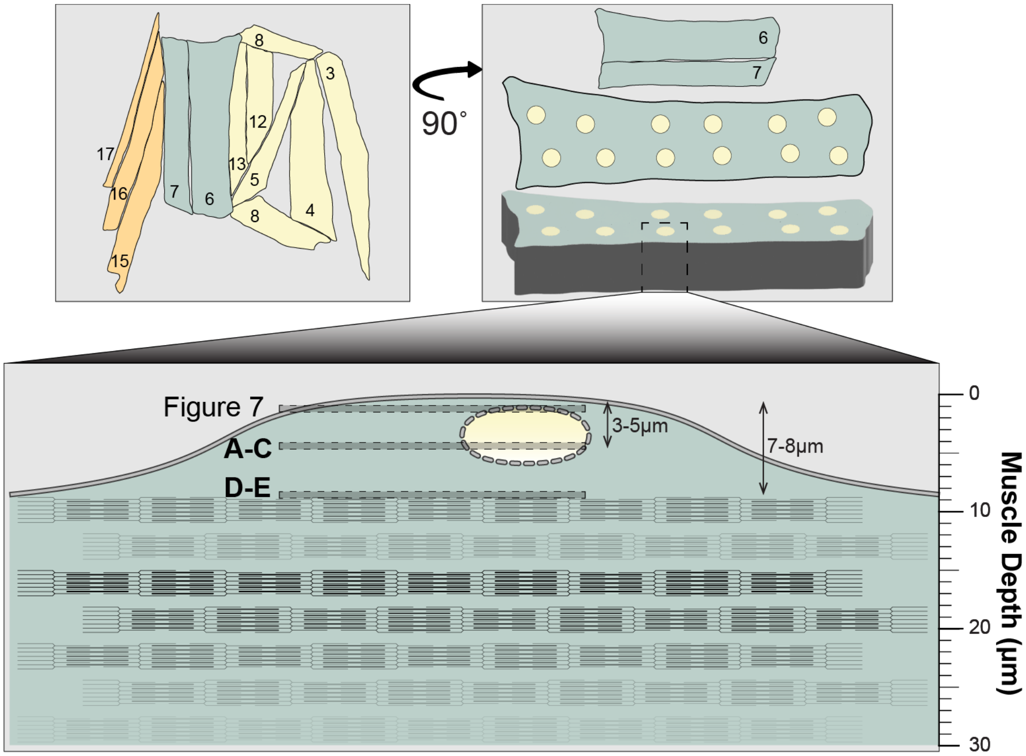

MSP300-GFP/+;24B>tdTom-Sec61β

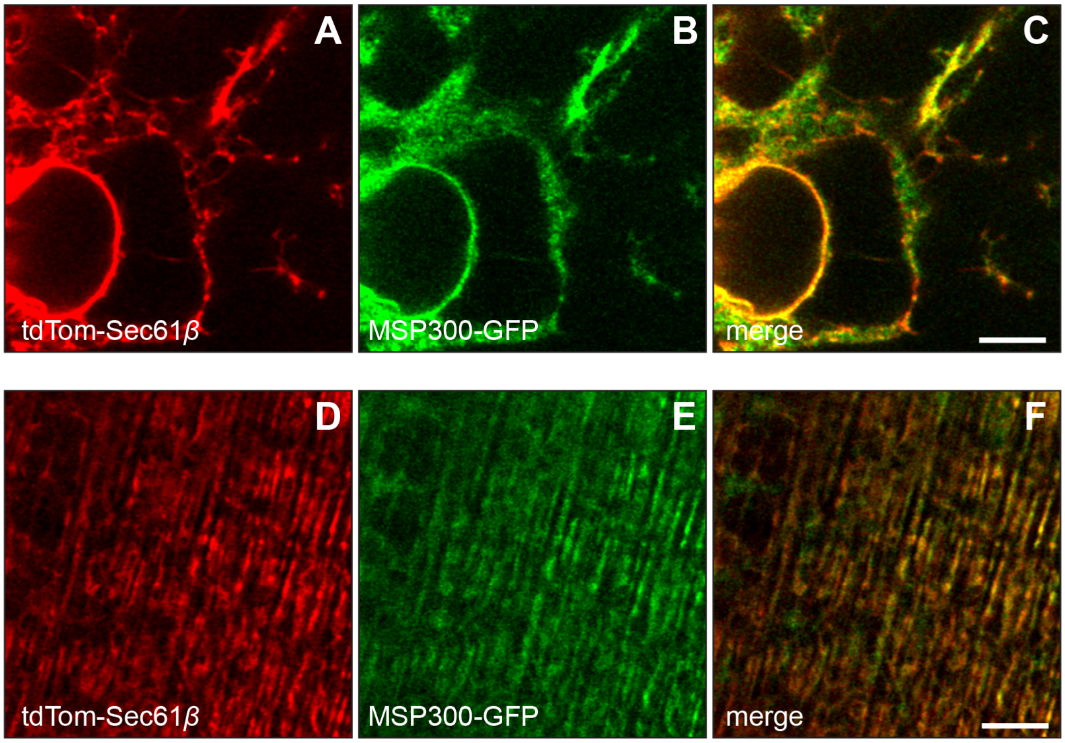

Fig. S5. MSP300 occupies the entire Endoplasmic Reticulum. (A). (Top) Schematic representation of one hemisegment of larval musculature with muscles labeled. The body wall muscles studied in this work are the ventral lateral longitudinal muscles (VL, labeled 6, and 7, colored light blue). Muscle 6, rotated 90 degrees in X-Y plane, is indicated in isolation with multiple nuclei indicated as yellow circles. A 90˚ rotation in the Z-dimension is shown to indicate the relative depth of this larval body wall muscle. A higher resolution cartoon of an isolated portion of muscle 6 is shown indicating a protrusion of the cytoplasm in Z-plane to accommodate the nucleas (shown in yellow). This portion of the schematic if drawn roughly to scale indicated by the ruler on the right (in μm). Confocal optical section approximately 25 x 25x 0.6μm are shown as dashed transparent grey boxes. The section shown in Figure 7 is located most medially (closest to the hemolymph in the intact larvae). Panels A-C and D-F shown below are also indicated relative to the medial surface of the muscle.

Live confocal imaging (maximum intensity z-projections) of larval body wall muscle 6 expressing the resident ER marker protein, tdTomato-Sec61β (**A, D**, tdTom, red) and MSP300-EFGP (**B, E**, EGFP, green) taken from focal plane indicated above. **(C, F)** Merged image of tdTomato and EGFP. Scale bar equals 5 μm.

Journal of Cell Science • Supplementary information
