# Supplementary material for: The Drosophila Nesprin-1 homolog MSP300 is required for muscle autophagy and proteostasis
Source: J Cell Sci. 2024 Jun 10;137(11):jcs262096. doi: 10.1242/jcs.262096 (PMC11213522; doi:10.1242/jcs.262096)
Supplement: Supplementary file 1 [file joces-137-262096_review_history.pdf]

The *Drosophila* Nesprin-1 homolog MSP300 is required for muscle autophagy and proteostasis

Kevin van der Graaf, Saurabh Srivastav, Rajkishor Nishad, Michael Stern and James A. McNew

DOI: 10.1242/jcs.262096

Editor: Giampietro Schiavo

Review timeline
Original submission: 6 March 2024
Editorial decision: 24 April 2024
First revision received: 30 April 2024
Accepted: 13 May 2024

Original submission

First decision letter

MS ID#: JOCES/2024/262096

MS TITLE: The Drosophila Nesprin-1 homolog MSP300 is required for muscle autophagy and proteostasis

AUTHORS: Kevin van der Graaf, Saurabh Srivastav, Rajkishor Nishad, Michael Stern, and James A McNew

We have now reached a decision on the above manuscript.

To see the reviewers' reports and a copy of this decision letter, please go to: https://submit-jcs.biologists.org and click on the 'Manuscripts with Decisions' queue in the Author Area. (Corresponding author only has access to reviews.)

As you will see, the reviewers raise several substantial criticisms that prevent me from accepting the paper at this stage. They suggest, however, that a revised version might prove acceptable, if you can address their concerns. If you think that you can deal satisfactorily with the criticisms on revision, I would be pleased to see a revised manuscript. We would then return it to the reviewers.

Please ensure that you clearly highlight all changes made in the revised manuscript. Please avoid using 'Tracked changes' in Word files as these are lost in PDF conversion.

I should be grateful if you would also provide a point-by-point response detailing how you have dealt with the points raised by the reviewers in the 'Response to Reviewers' box. Please attend to all of the reviewers' comments. If you do not agree with any of their criticisms or suggestions please explain clearly why this is so.

Reviewer 1

Advance summary and potential significance to field

© 2024. Published by The Company of Biologists under the terms of the Creative Commons Attribution License (https://creativecommons.org/licenses/by/4.0/).

The present manuscript analyzes the involvement of MSP300, a Nesprin homolog, in muscle autophagy and proteostasis. The authors analysis reveal interesting and novel functions for Nesprin/MSP300 in the cytoplasm, which presumably are unrelated to its nuclear functions. They show that MSP300 knockdown leads to accumulation of cytoplasmic aggregates containing poly-ubiquinated cargo, in combination with the autophagy receptor ref(2)P/p62/SQSTM, as well as with Atg8a suggesting a specific role for MSP300 in autophagy, possibly in autophagosome maturation. Because MSP300 localizes also to the ER the authors suggest that MSP300 might affect the completion of autophagosome through a role in controlling cytoskeletal contact sites. While this last possibility is highly speculative, it is consistent with many previous observations. The paper adds to our knowledge on MSP300 function in muscle cells, and consequently provides an additional explanation for human diseases associated with aberrant function of the LINC complex. One caveat with the description of these phenotypes is that the overall distribution of many organelles in the cytoplasm of muscles mutant for MSP300, including also the microtubule (MT) network, are severely defected in the mutants, so it is difficult to discriminate between primary and secondary phenotypes. Previously, defects in proper organization and distribution of organelles such as mitochondria, ER network, MT, were described (Elhanany-Tamir et al, 2012), implicating already abnormal distribution of organelles in the mutants. It is therefore crucial to discriminate between primary and secondary phenotypes of the mutant muscle.

Comments for the author

The present manuscript analyzes the involvement of MSP300, a Nesprin homolog, in muscle autophagy and proteostasis. The authors analysis reveal interesting and novel functions for Nesprin/MSP300 in the cytoplasm, which presumably are unrelated to its nuclear functions. They show that MSP300 knockdown leads to accumulation of cytoplasmic aggregates containing poly-ubiquinated cargo, in combination with the autophagy receptor ref(2)P/p62/SQSTM, as well as with Atg8a suggesting a specific role for MSP300 in autophagy, possibly in autophagosome maturation. Because MSP300 localizes also to the ER the authors suggest that MSP300 might affect the completion of autophagosome through a role in controlling cytoskeletal contact sites. While this last possibility is highly speculative, it is consistent with many previous observations. The paper adds to our knowledge on MSP300 function in muscle cells, and consequently provides an additional explanation for human diseases associated with aberrant function of the LINC complex. One caveat with the description of these phenotypes is that the overall distribution of many organelles in the cytoplasm of muscles mutant for MSP300, including also the microtubule (MT) network, are severely defected in the mutants, so it is difficult to discriminate between primary and secondary phenotypes. Previously, defects in proper organization and distribution of organelles such as mitochondria, ER network, MT, were described (Elhanany-Tamir et al, 2012), implicating already abnormal distribution of organelles in the mutants. It is therefore crucial to discriminate between primary and secondary phenotypes of the mutant muscle.
Major comment:
1. Perform conditional knockdown of MSP300, using the RNAi line and a combination of Tub-Gal80ts and Mef2Gal4, and to induce knockdown during larval stages so that the authors be able to follow the phenotypes one after the other.
2. What is the evidence that MSP300 impacts on “proteostasis”, as indicated in the title of the manuscript.

Minor comments:
1. Figure 1 – The florescent signal is not distributed equally and it is difficult to identify the nuclei. Please improve the image.
2. How many larvae were analyzed?
3. What is the significance of large versus small aggregates?
4. Page 15, last line: Z-disc localization of MSP300 is detected by isoform B which might not be detected by the GFP trap used.

Reviewer 2

Advance summary and potential significance to field

Nesprins are nuclear enveloppe associated proteins which, together with SUN proteins, form the core of the LInker of Nucleus and Cytoskeleton complex (LINC) and play critical roles in nuclear

© 2024. Published by The Company of Biologists under the terms of the Creative Commons Attribution License (https://creativecommons.org/licenses/by/4.0/).

positionning and nuclear shape maintenance. Mutations in Nesprins are associated with numerous pathologies affecting muscles or neurons but the mechanisms underlying these pathologies are not fully understood.
In this paper, van der Graaf et al. describe the induction of autophagy in Drosophila larval muscle upon depletion of Msp300, Drosophila Nesprin ortholog.
Nesprins contain a KASH domain which anchors them in the nuclear enveloppe. Deletion of the KASH of Msp300 or depletion of Msp300 by RNAi in larval muscle have been previously shown to induce nuclei clustering and alteration of muscle organisation.
Here the authors first compare 4 conditions of Msp300 alteration : the KASH domain deletion and RNAi depletion using three muscle specific drivers of increasing strength, two leading to muscle damages and pupal death. In all conditions they observed nuclei clustering as previously published as well as accumulation of polyUbiquitinated proteins, a sign of impaired autophagy.
They then go on characterising the autophagy deficit, focussing on a mild RNAi condition (Mhc driver), proceeding similarly to their previous work on atlastin (Srivastav et al. 2024).
They first classify polyUb aggregates in three categories based on their size and show an increase in polyUb aggregates in all categrories upon Msp300 RNAi. These aggregates are labelled with two markers of autophagosomes : p62, which labels the vast majority of median and large aggregates, and Atg8a which only labels 50% of these aggregates.
The authors overexpressed a chimeric mCherry-Atg8a-GFP protein, commonly used to assess fusion of autophagosomes to lysosomes. They report that increased expression of Atg8a improved the labelling of polyUb aggregates but did not restore autophagy flux. mCherry-Atg8a-GFP is used as a read-out of autophagy progression to fusion with lysosomes as GFP is quenched in acidic environment. Msp300 RNAi larvae show an increase in the % of clusters co-labelled for GFP and mCherry, signing a deficit in autophagy progression.
Last, as Msp300RNAi phenotypes are similar to the one described for atlastin depletion, the authors investigate a potential contribution of ER. They show that Msp300 decorates the ER and that Msp300 RNAi leads to an increase in the activation of FOXO, an ER stress marker. Notably, they describe here a high variability in FOXO activation between the muscle studied and the individuals.

Comments for the author

Major comments :
1- van der Graaf et al. present here a very descriptive work of a link between Msp300 and autophagy but do not propose any molecular mechanism. Although a link between Msp300 and autophagy in Drosophila has not been shown before, Nesprins have already been shown to contribute to the progression of autophagy in C. elegans and in mammalian Mefs cells (Papandreou et al. 2023, DOI: 10.1038/s43587-022-00327-4). Furthermore, Kucinska et al. (2023, DOI : 10.1038/s43587-022-00327-4) showed that resolution of ER stress by autophagy involves the disassembly of the SUN/Nesprin LINC complex at the nuclear enveloppe. As such, this work is neither new nor does it provide mechanistic insights into the role of Msp300/Nesprin in autophagy.

2- I also have a major concern regarding the sample nature and size used in these experiments. All the data reported here come from the analysis of 6 larvae and 3 muscles per larvae (muscle 6 from abdominal segments 2 to 4). All quantifications however reveal a hight variability between individuals, specifically in the mutant condition. This variability is clearly stated in Figure 6 with the quantification of Foxo activity for which the authors report a strong variability between abdominal segments but also between individuals. Considering this variability the sample size used here is not sufficient.
The sample size needs to be rationalized by measuring one muscle per larva (always from the same abdominal segment) and increasing the number of larvae assessed to at least 10-15.
Similarly, in Fig S2, if all damaged muscle on panel I correspond to 2 larvae only and the normal one in panel F to a single one, it should be stated.
Last, considering the sample size, the statistics used here are inappropriate and the authors should use non-parametric statistics such as Wilcoxon test. As well, unless sample size is larger than 30, median and quartile should be used in all figures.

3- Kucinska et al. (2023, DOI : 10.1038/s43587-022-00327-4) showed that resolution of ER stress by autophagy involves the disassembly of the SUN/Nesprin LINC complex at the nuclear enveloppe. Here, the authors make several observations in agreement with such role in Drosophila : first they

© 2024. Published by The Company of Biologists under the terms of the Creative Commons Attribution License (https://creativecommons.org/licenses/by/4.0/).

observe that “larger aggregates tended to localize near the clustered nuclei” (Fig 2). They further report the co-localisation of Msp300 and the ER protein sec61b (Fig7) and speculate on the role of Msp300 in ER dynamics. The consequence of Msp300 RNAi on ER morphology, and specifically in the vicinity of clustered nuclei, is clearly missing to address the relatioship between ER, Nuclear envelope and Msp300 and needs to be investigated.

Minor comments:
1- Figure 1-3, the contrast is not very good, making it difficult to identify the muscles. Muscles effectively measured should be stated and outlined. As far as I understand, measured are done of muscle 6.

2- Figure 3 , the author characterize the polyUB aggregate phenotype upon Msp300 RNAi depletion. In panel N, the measures for the control are missing.

3- Figure 6, both muscle 6 and 7 are outlined together. They should be outlined separately. Is the quantification performed on muscle 6 only or both 6 and 7. Indeed, as LacZ intensity seems much weaker on muscle 7 than 6 (6B), measures should all be done on muscle 6.

4- Figure 6 D,E, reveal a high variability between segments and individuals. To which extend does this variability reflect inter individual variability? Please, state which measures correspond to the same larva.

5- P12, 70% of the polyUB aggregates are positive for Atg8a in mhc>Msp300; GFP-mCherry-atg8a (Fig 4G) and not 70% negative as writen.

6- The authors fail to observe Z-disc localization with the protein trap between exon 33 and 34. This trap is expected to tag the isoforms D, G-M but neither B, F or E. In contrast, in the previous reports showing a Z-Disc localisation the authors used either a YFP line tagging the B, D, L,H, I-K but not EG and M isoforms or an anti Msp300 antibody directed against a domain in the Nter half of the protein. The possibility that the absence of Z-disc labeling rather reflects an isoform specificity should thus also be mentionned.

Reviewer 3

Advance summary and potential significance to field

The transmembrane protein MSP300 is known to localize to the nuclear membrane and bridge interactions with chromatin and cytosolic structural components through interactions with proteins spanning the inner membrane.

Here, the authors provide evidence for a novel function for MSP300: clearing ubiquitinated cargo tagged for autophagic degradation. Analysis of autophagy flux markers suggests that the defect is upstream of auophagosome-lysosome fusion.

As MSP300 is localized to the ER, where it may be part of the growing list of proteins involved in organelle contact sites controlling autophagy and other vesicle trafficking processes.

Comments for the author

In the submitted work, de Graaf et al. purport that the Drosophila Nesprin-1 homologue MSP300 is required for autophagy flux and proteostasis.

The transmembrane protein MSP300 is known to localize to the nuclear membrane and bridge interactions with chromatin and cytosolic structural components through interactions with proteins spanning the inner membrane. Loss of function (LOF) studies of MSP300 in flies is known to cause nuclear mispositioning in the larval muscle syncytium. Here, the authors report additional findings to suggest that MSP300 is required for autophagy flux/proteostasis. Using a combination of immunolabeling and a GFP-mCherry tagged Atg8a autophagy marker that can distinguish autophagosomes or other structures before and after lysosome fusion the authors conclude that

© 2024. Published by The Company of Biologists under the terms of the Creative Commons Attribution License (https://creativecommons.org/licenses/by/4.0/).

autophagy flux to the lysosome is defective. Assessing the 4EBP/Thor marker expression suggests that MSP300 knockdown leads to reduced growth signaling, likely due to reduced TOR1 and/or increased Foxo activity, which should stimulate autophagy initiation, but this is not pursued further. Tagged MSP300 additionally localizes to the endoplasmic reticulum—which is contiguous with the nuclear membrane. Similarities in autophagy defects upon Atlastin LOF, a protein involved in ER fusion events lead the authors to speculate that the autophagy defect may be due to a hitherto undefined ER resident role for MSP300 in autophagy.This is not further adressed.

Whereas the accumulation of Ubiquitin/Ref(2)P/p62/Atg8a and lack of flux of double-tagged GFP-mCherry-Atg8a is consistent with a defect in autophagy and consequently protein aggregate accumulation, a number of experiments should be performed to make this conclusion and the title of the paper sound and warranted.

* While accumulation of Ub positive structures could be ubiquitinated protein aggregates that recruit the autophagy adapter protein p62/Ref(2)P, no other experiments address if this is the case. Other alternatives include organelles for instance mitochondria or cell remnants acquired during cell fusion during muscle syncytium formation.
To address this:
* Perform Western Blot experiments to address whether ubiquitinated protein aggregates do form. They should appear as a smear by probing Western Blots by anti Ub.
* Perform ultrastructural studies by immuno-EM or CLEM (Correlative Light and Electron Microscopy) to define the nature of the large Ub/Atg8a positive structures. Do the autophagy markers label protein aggregates, organelles aggregated autophagosomes. -EM would also help address if autophagy is impaired and at which stage.
* To address if autophagy flux depends on MSP300, a starvation-induced pulse-chase experimental regime in the fat body starvation responsive tissue would bolster evidence.
* Western Blot analysis to address if tagged Atg8a is prohibited from reaching the lysosome can be used to support defects in autophagy flux upon MSP300 knockdown.

Specific comments
Fig 1. For the legend and elsewhere in the manuscript, please specify which of the two TRiP lines was used for MSP300 knockdown experiments.
It is argued that nuclear clustering severity closely follows that of muscle defects (thin muscle fibers, Supplementary Fig 2). Why not include the DeltaKash homozygous animals? It would add useful information in terms of structure-function analysis. Is muscle thinning seen in the attP40 "landing site" lines as well? Refer to Supplementary Fig 3. This would be informative to the field as a great number of RNAi lines carry these landing sites and they would carry significant experimental caveats.

Fig 2 Ubiquitin-positive puncta increase in muscles deficient for MSP300 presumably representing protein aggregates. It would be important to see if this phenotype extends to other tissues, like diploid imaginal disc cells, to settle if this phenotype is related to multinucleated cells. A simple compartmental knockdown using en-gal4, ap-Gal4 or similar drivers in wing discs can be easily performed to this end. A western blot probed for total protein and poly-ubiquitin of deltaKASH mutant animals and upon muscle knockdown must be performed to support the assumption that ubiquitinated proteins accumulate in mutant tissues, i.e., that proteostasis is defective. General accumulation of ubiquitinated proteins should appear as a smear by Western Blot.

Fig 3 Most Ub positive aggregates colocalize with p62/ref(2)P, known to tag ubiquitinated proteins for autophagic capture and degradation. In agreement with this logic, many Ub puncta are also positive for Atg8a by immunostaining. The authors note that the larger aggregates stain relatively less well for Atg8a whereas it is labeled by the GFP-Atg8 transgene. The authors may want to consider that one possibility for this discrepancy may be that GFP-Atg8a is incorporated as the aggregates form, while antibody staining will stain more easily at the surface. This has been previously observed in several studies.

Section: MSP300 reduction led to impaired autophagic flux and to the accumulation of undegraded cargo.

© 2024. Published by The Company of Biologists under the terms of the Creative Commons Attribution License (https://creativecommons.org/licenses/by/4.0/).

The authors utilize double-tagged GFP-mCherry-Atg8a to assess a defect in autophagic flux. It is true that an increase in double-labeled structures can be indicative of reduced autophagic flux. As this is the central finding of the paper, this hypothesis should be supported by a more direct measure of autophagic flux. One option is to measure autophagic flux by quantifying the amount of tagged Atg8a reaching the lysosome by Western Blot. When GFP or mCherry-tagged Atg8a reaches the lysosome, GFP/mCherry is freed from Atg8a by lysosomal digestion. As both GFP and mCherry are moderately resistant to lysosomal digestion, it can easily be assessed by Western Blot analysis and distinguished from tagged Atg8a due to a shift in size. The amount of mCherry or GFP "alone" is considered a measure of autophagic flux and represents a readily available way to measure autophagy flux in vivo using the tools and experimental regime in the paper.

Figure 5
How was particle volume calculated?
The accumulation of large GFP and Cherry-Atg8a structures that colocalize with polyUb suggests a defective autophagic flux. It would be highly informative to provide EM images, preferably by CLEM or immuno-EM (using anti GFP, Cherry or PolyUb) to diagnose what these structures are. Are the large accumulations of autophagy vesicles, protein aggregates, or some other structures related to muscle formation? This would help identify at what step in autophagic flux MSP300 plays a role.

To assess whether MSP300 indeed is required for autophagy flux, the authors should resort to the nutrient-responsive tissue, the fat body. Using tagged GFP-mCherry-Atg8a, the authors can easily perform controlled pulse chase experiments and resolve whether MSP300 is required for autophagy flux with time resolution within a few hours by confocal microscopy. This experiment would have the benefit of extending findings to a non-muscle tissue to exclude functions related to myoblast fusion, for instance. Please consult PMID: 24554766 for instance on how autophagy-lysosome fusion defect would manifest phenotypically and how to perform pulse chase assays.

Minor: Stick to one spelling. PolyUb or PolyUB

Fig 6 Increased 4EBP/Thor expression indicative of reduced PI3K-TORC1 growth signaling. This would in principle increase autophagy flux and reduce growth consistent with the thinner fibers upon MSP300 but is pursued further as the manuscript now stands.

Fig 7. Colocalization experiment of an ER marker and EGFP-tagged MSP300 reveal ER localization, which is interesting, but a bit tangential to the main findings of the paper without further experiments in the direction to show that this location is where autophagy is initiated for instance. Colocalization experiments between Cherry-Atg8a and EGFP-MSP300 upon a starvation regime would be a starting point for such an avenue of experimentation. Some obvious controls are lacking, such as expression of either marker alone to see if they affect each other.

First revision

Author response to reviewers' comments

Reviewer 1 Advance summary and potential significance to field
The present manuscript analyzes the involvement of MSP300, a Nesprin homolog, in muscle autophagy and proteostasis. The authors analysis reveal interesting and novel functions for Nesprin/MSP300 in the cytoplasm, which presumably are unrelated to its nuclear functions. They show that MSP300 knockdown leads to accumulation of cytoplasmic aggregates containing poly-ubiquinated cargo, in combination with the autophagy receptor ref(2)P/p62/SQSTM, as well as with Atg8a, suggesting a specific role for MSP300 in autophagy, possibly in autophagosome maturation. Because MSP300 localizes also to the ER the authors suggest that MSP300 might affect the completion of autophagosome through a role in controlling cytoskeletal contact sites. While this last possibility is highly speculative, it is consistent with many previous observations. The paper adds to our knowledge on MSP300 function in muscle cells, and consequently provides an additional explanation for human diseases associated with aberrant function of the LINC complex. One caveat with the description of these phenotypes is that the overall distribution of many organelles in the

© 2024. Published by The Company of Biologists under the terms of the Creative Commons Attribution License (https://creativecommons.org/licenses/by/4.0/).

cytoplasm of muscles mutant for MSP300, including also the microtubule (MT) network, are severely defected in the mutants, so it is difficult to discriminate between primary and secondary phenotypes. Previously, defects in proper organization and distribution of organelles such as mitochondria, ER network, MT, were described (Elhanany-Tamir et al, 2012), implicating already abnormal distribution of organelles in the mutants. It is therefore crucial to discriminate between primary and secondary phenotypes of the mutant muscle.

Reviewer 1 Comments for the author

The present manuscript analyzes the involvement of MSP300, a Nesprin homolog, in muscle autophagy and proteostasis. The authors analysis reveal interesting and novel functions for Nesprin/MSP300 in the cytoplasm, which presumably are unrelated to its nuclear functions. They show that MSP300 knockdown leads to accumulation of cytoplasmic aggregates containing poly-ubiquinated cargo, in combination with the autophagy receptor ref(2)P/p62/SQSTM, as well as with Atg8a, suggesting a specific role for MSP300 in autophagy, possibly in autophagosome maturation. Because MSP300 localizes also to the ER the authors suggest that MSP300 might affect the completion of autophagosome through a role in controlling cytoskeletal contact sites. While this last possibility is highly speculative, it is consistent with many previous observations. The paper adds to our knowledge on MSP300 function in muscle cells, and consequently provides an additional explanation for human diseases associated with aberrant function of the LINC complex. One caveat with the description of these phenotypes is that the overall distribution of many organelles in the cytoplasm of muscles mutant for MSP300, including also the microtubule (MT) network, are severely defected in the mutants, so it is difficult to discriminate between primary and secondary phenotypes. Previously, defects in proper organization and distribution of organelles such as mitochondria, ER network, MT, were described (Elhanany-Tamir et al, 2012), implicating already abnormal distribution of organelles in the mutants. It is therefore crucial to discriminate between primary and secondary phenotypes of the mutant muscle.

Major comment:
1.Perform conditional knockdown of MSP300, using the RNAi line and a combination of Tub-Gal80ts and Mef2Gal4, and to induce knockdown during larval stages so that the authors be able to follow the phenotypes one after the other.

We agree that this is an excellent experiment and would be very informative. However, we view this experiment, and other related experiments, as opening up new avenues of exploration for this phenomenon rather than as completion of existing avenues, and therefore believe that this experiment is more appropriate for subsequent studies.

2.What is the evidence that MSP300 impacts on “proteostasis”, as indicated in the title of the manuscript.

We use the term to indicate proper maintenance of the proteome over time. With this definition, the accumulation of undegraded, ubiquitinated cargo, GFP signal within autophagosomes and cell degeneration all indicate failure of proteostasis in muscle lacking MSP300.

Minor comments:
1.Figure 1 – The florescent signal is not distributed equally and it is difficult to identify the nuclei. Please improve the image.

This issue appears to be caused by the properties of the larval muscle. The larval muscles are not flat, but rather the surface undulates, which makes it difficult to acquire even fluorescence across the entire surface.

2.How many larvae were analyzed?

Six larvae per genotype.

3.What is the significance of large versus small aggregates?

© 2024. Published by The Company of Biologists under the terms of the Creative Commons Attribution License (https://creativecommons.org/licenses/by/4.0/).

We are not sure, but our working hypothesis is that the larger aggregates represent aggregates that formed early, whereas the smaller aggregates formed later. Further, we think that as the larger aggregates grow, at some point they grow too large for proper processing through autophagy, which is why these larger aggregates accumulate specifically in autophagy-crippled genotypes. We would prefer not to discuss this working hypothesis in detail in the paper, as these thoughts are extremely speculative.

4.Page 15, last line: Z-disc localization of MSP300 is detected by isoform B which might not be detected by the GFP trap used.

We amended the text to address this issue and are grateful to this reviewer for pointing out this explanation.

*****
Reviewer 2 Advance summary and potential significance to field
Nesprins are nuclear enveloppe associated proteins which, together with SUN proteins, form the core of the LInker of Nucleus and Cytoskeleton complex (LINC) and play critical roles in nuclear positionning and nuclear shape maintenance. Mutations in Nesprins are associated with numerous pathologies affecting muscles or neurons but the mechanisms underlying these pathologies are not fully understood.
In this paper, van der Graaf et al. describe the induction of autophagy in Drosophila larval muscle upon depletion of Msp300, Drosophila Nesprin ortholog.
Nesprins contain a KASH domain which anchors them in the nuclear enveloppe. Deletion of the KASH of Msp300 or depletion of Msp300 by RNAi in larval muscle have been previously shown to induce nuclei clustering and alteration of muscle organisation.
Here the authors first compare 4 conditions of Msp300 alteration : the KASH domain deletion and RNAi depletion using three muscle specific drivers of increasing strength, two leading to muscle damages and pupal death. In all conditions they observed nuclei clustering as previously published as well as accumulation of polyUbiquitinated proteins, a sign of impaired autophagy.
They then go on characterising the autophagy deficit, focussing on a mild RNAi condition (Mhc driver), proceeding similarly to their previous work on atlastin (Srivastav et al. 2024).
They first classify polyUb aggregates in three categories based on their size and show an increase in polyUb aggregates in all categrories upon Msp300 RNAi. These aggregates are labelled with two markers of autophagosomes : p62, which labels the vast majority of median and large aggregates, and Atg8a which only labels 50% of these aggregates.
The authors overexpressed a chimeric mCherry-Atg8a-GFP protein, commonly used to assess fusion of autophagosomes to lysosomes. They report that increased expression of Atg8a improved the labelling of polyUb aggregates but did not restore autophagy flux. mCherry-Atg8a-GFP is used as a read-out of autophagy progression to fusion with lysosomes as GFP is quenched in acidic environment. Msp300 RNAi larvae show an increase in the % of clusters co-labelled for GFP and mCherry, signing a deficit in autophagy progression.
Last, as Msp300RNAi phenotypes are similar to the one described for atlastin depletion, the authors investigate a potential contribution of ER. They show that Msp300 decorates the ER and that Msp300 RNAi leads to an increase in the activation of FOXO, an ER stress marker. Notably, they describe here a high variability in FOXO activation between the muscle studied and the individuals.

Reviewer 2 Comments for the author
Major comments :
1- van der Graaf et al. present here a very descriptive work of a link between Msp300 and autophagy but do not propose any molecular mechanism. Although a link between Msp300 and autophagy in Drosophila has not been shown before, Nesprins have already been shown to contribute to the progression of autophagy in C. elegans and in mammalian Mefs cells (Papandreou et al. 2023, DOI: 10.1038/s43587-022-00327-4). Furthermore, Kucinska et al. (2023, DOI : 10.1038/s43587-022-00327-4) showed that resolution of ER stress by autophagy involves the disassembly of the SUN/Nesprin LINC complex at the nuclear enveloppe. As such, this work is neither new nor does it provide mechanistic insights into the role of Msp300/Nesprin in autophagy.

© 2024. Published by The Company of Biologists under the terms of the Creative Commons Attribution License (https://creativecommons.org/licenses/by/4.0/).

We are grateful to this reviewer for pointing out the Papandreou paper and have added this reference and text to the introduction (last sentence, third paragraph). The other references did not seem to us to be relevant to our studies. However, we respectfully disagree that this work is "neither new nor does it provide mechanistic insights.........". Our work has the following novel features, not shown in Papandreou et al.; first, we showed that GFP-tagged Atg8a retains GFP signal, indicating that autophagy failure appears to occur at a late stage in autophagy, second, that the ΔKASH mutation shows autophagy defects similar to the knockdown, indicating that direct membrane tethering of MSP300 is required for autophagy activity, third, that muscle degeneration occurs in strong MSP300 knockdown, fourth, that the stress response transcription factor Foxo is activated in MSP300 knockdown larvae, fifth, whereas Atg8a is limiting within large polyUB aggregates, Atg8a overexpression fails to rescue the autophagy defect, and sixth, MSP300 is located to the ER, which provides mechanistic possibilities as to its role in autophagy.

2- I also have a major concern regarding the sample nature and size used in these experiments. All the data reported here come from the analysis of 6 larvae and 3 muscles per larvae (muscle 6 from abdominal segments 2 to 4). All quantifications however reveal a hight variability between individuals, specifically in the mutant condition. This variability is clearly stated in Figure 6 with the quantification of Foxo activity for which the authors report a strong variability between abdominal segments but also between individuals. Considering this variability the sample size used here is not sufficient.
The sample size needs to be rationalized by measuring one muscle per larva (always from the same abdominal segment) and increasing the number of larvae assessed to at least 10-15.

The p-values we report for our statistically significant results are very low, often <0.0001, so we fail to see the value in increasing the sample size further. In addition, sample size needs to be selected in advance and it is improper to adjust sample size after an experiment has begun.

Similarly, in Fig S2, if all damaged muscle on panel I correspond to 2 larvae only and the normal one in panel F to a single one, it should be stated.
Last, considering the sample size, the statistics used here are inappropriate and the authors should use non-parametric statistics such as Wilcoxon test. As well, unless sample size is larger than 30, median and quartile should be used in all figures.

We (and others) have published many papers involving statistical analysis of polyUB size and number in Drosophila larval muscles. If we switched our method of statistical analysis, it would be very difficult for readers to compare these new results with previously published results. In contrast, maintaining the same statistical approach in this paper to previous papers enables the reader to easily compare these new results with relevant data in previous papers.

3- Kucinska et al. (2023, DOI : 10.1038/s43587-022-00327-4) showed that resolution of ER stress by autophagy involves the disassembly of the SUN/Nesprin LINC complex at the nuclear enveloppe. Here, the authors make several observations in agreement with such role in Drosophila : first they observe that “larger aggregates tended to localize near the clustered nuclei” (Fig 2). They further report the co-localisation of Msp300 and the ER protein sec61b (Fig7) and speculate on the role of Msp300 in ER dynamics. The consequence of Msp300 RNAi on ER morphology, and specifically in the vicinity of clustered nuclei, is clearly missing to address the relatioship between ER, Nuclear envelope and Msp300 and needs to be investigated.

We will be presenting these results in a different study in which we will report the effects of several different perturbations, including MSP300, on ER structure.

Minor comments:
1-Figure 1-3, the contrast is not very good, making it difficult to identify the muscles. Muscles effectively measured should be stated and outlined. As far as I understand, measured are done of muscle 6.

We apologize that the image quality of the PNG files used for the creation of the combined PDF required for initial submission. This issue should be corrected now that native EPS files are available for inspection.

© 2024. Published by The Company of Biologists under the terms of the Creative Commons Attribution License (https://creativecommons.org/licenses/by/4.0/).

2-Figure 3 , the author characterize the polyUB aggregate phenotype upon Msp300 RNAi depletion. In panel N, the measures for the control are missing.

The focus here is on the larger sized aggregates, which are so infrequent in wildtype that it is not meaningful to employ statistical analysis. Therefore, here we are comparing occupancy of p62 and Atg8a only for the MSP300 knockdown.

3-Figure 6, both muscle 6 and 7 are outlined together. They should be outlined separately. Is the quantification performed on muscle 6 only or both 6 and 7. Indeed, as LacZ intensity seems much weaker on muscle 7 than 6 (6B), measures should all be done on muscle 6.

Prior to conducting the experiment, we decided to count nuclear beta-gal intensity in both muscles 6 and 7, which is what we did. Since we included both muscles in out count, we outlined both muscles together.

4-Figure 6 D,E, reveal a high variability between segments and individuals. To which extend does this variability reflect inter individual variability? Please, state which measures correspond to the same larva.

Correct, and it can be seen in Figure 6B that there is extensive variability of nuclear 4E-BP even within the same muscle. We don't understand this behavior. We have tried to correlate intensity of nuclear signal with properties such as presence within a nuclear cluster, nuclear caging by microtubules, etc., but did not find any significant correlations.

5-P12, 70% of the polyUB aggregates are positive for Atg8a in mhc>Msp300; GFP-mCherry-atg8a (Fig 4G) and not 70% negative as writen.

Not exactly. The smallest puncta have ~30% puncta positive for Atg8a, and these dominant the polyUB number, as the vast majority of polyUB puncta are small (see Figure 4F).

6-The authors fail to observe Z-disc localization with the protein trap between exon 33 and 34. This trap is expected to tag the isoforms D, G-M but neither B, F or E. In contrast, in the previous reports showing a Z-Disc localisation the authors used either a YFP line tagging the B, D, L,H, I-K but not EG and M isoforms or an anti Msp300 antibody directed against a domain in the Nter half of the protein. The possibility that the absence of Z-disc labeling rather reflects an isoform specificity should thus also be mentionned.

See reviewer #1 comment and response above.

*****

Reviewer 3 Advance summary and potential significance to field
The transmembrane protein MSP300 is known to localize to the nuclear membrane and bridge interactions with chromatin and cytosolic structural components through interactions with proteins spanning the inner membrane.

Here, the authors provide evidence for a novel function for MSP300: clearing ubiquitinated cargo tagged for autophagic degradation. Analysis of autophagy flux markers suggests that the defect is upstream of auophagosome-lysosome fusion.

As MSP300 is localized to the ER, where it may be part of the growing list of proteins involved in organelle contact sites controlling autophagy and other vesicle trafficking processes.

Reviewer 3 Comments for the author
In the submitted work, de Graaf et al. purport that the Drosophila Nesprin-1 homologue MSP300 is required for autophagy flux and proteostasis.

The transmembrane protein MSP300 is known to localize to the nuclear membrane and bridge interactions with chromatin and cytosolic structural components through interactions with proteins spanning the inner membrane. Loss of function (LOF) studies of MSP300 in flies is known to cause nuclear mispositioning in the larval muscle syncytium. Here, the authors report additional findings

© 2024. Published by The Company of Biologists under the terms of the Creative Commons Attribution License (https://creativecommons.org/licenses/by/4.0/).

to suggest that MSP300 is required for autophagy flux/proteostasis. Using a combination of immunolabeling and a GFP-mCherry tagged Atg8a autophagy marker that can distinguish autophagosomes or other structures before and after lysosome fusion, the authors conclude that autophagy flux to the lysosome is defective. Assessing the 4EBP/Thor marker expression suggests that MSP300 knockdown leads to reduced growth signaling, likely due to reduced TOR1 and/or increased Foxo activity, which should stimulate autophagy initiation, but this is not pursued further. Tagged MSP300 additionally localizes to the endoplasmic reticulum—which is contiguous with the nuclear membrane. Similarities in autophagy defects upon Atlastin LOF, a protein involved in ER fusion events lead the authors to speculate that the autophagy defect may be due to a hitherto undefined ER resident role for MSP300 in autophagy. This is not further adressed.

Whereas the accumulation of Ubiquitin/Ref(2)P/p62/Atg8a and lack of flux of double-tagged GFP-mCherry-Atg8a is consistent with a defect in autophagy and consequently protein aggregate accumulation, a number of experiments should be performed to make this conclusion and the title of the paper sound and warranted.

* While accumulation of Ub positive structures could be ubiquitinated protein aggregates that recruit the autophagy adapter protein p62/Ref(2)P, no other experiments address if this is the case. Other alternatives include organelles, for instance mitochondria or cell remnants acquired during cell fusion during muscle syncytium formation.

We agree that mitochondria or other organelles could be present within these clusters.

To address this:
* Perform Western Blot experiments to address whether ubiquitinated protein aggregates do form. They should appear as a smear by probing Western Blots by anti Ub.

The standard in the field is to use various imaging protocols rather than Western blotting to study polyUB aggregates.

* Perform ultrastructural studies by immuno-EM or CLEM (Correlative Light and Electron Microscopy) to define the nature of the large Ub/Atg8a positive structures. Do the autophagy markers label protein aggregates, organelles, aggregated autophagosomes. -EM would also help address if autophagy is impaired and at which stage.

Immuno-EM or CLEM analysis would be informative but are far beyond the scope of this paper, but we anticipate that the publication of this paper would inspire colleagues in the field to pursue such experiments.

* To address if autophagy flux depends on MSP300, a starvation-induced pulse-chase experimental regime in the fat body starvation responsive tissue would bolster evidence.

Studies of MSP300 effects in the fat body, either with or without starvation, is far beyond the scope of this paper, but we anticipate that the publication of this paper would inspire colleagues in the field to pursue such experiments.

* Western Blot analysis to address if tagged Atg8a is prohibited from reaching the lysosome can be used to support defects in autophagy flux upon MSP300 knockdown.

Given that Atg8a located within the autophagosome is degraded in the lysosome, we are not sure how this experiment would be informative.

Specific comments
Fig 1. For the legend and elsewhere in the manuscript, please specify which of the two TRiP lines was used for MSP300 knockdown experiments.

The identity of the TRiP line used in this study was described in the methods section.

It is argued that nuclear clustering severity closely follows that of muscle defects (thin muscle fibers, Supplementary Fig 2). Why not include the DeltaKash homozygous animals? It would add

© 2024. Published by The Company of Biologists under the terms of the Creative Commons Attribution License (https://creativecommons.org/licenses/by/4.0/).

useful information in terms of structure-function analysis. Is muscle thinning seen in the attP40 "landing site" lines as well? Refer to Supplementary Fig 3. This would be informative to the field as a great number of RNAi lines carry these landing sites and they would carry significant experimental caveats.

We described the effects of attP40 on many larval and adult phenotypes in our previous paper (van der Graaf et al., 2022, PMID 36516171).

Fig 2 Ubiquitin-positive puncta increase in muscles deficient for MSP300, presumably representing protein aggregates. It would be important to see if this phenotype extends to other tissues, like diploid imaginal disc cells, to settle if this phenotype is related to multinucleated cells. A simple compartmental knockdown using en-gal4, ap-Gal4 or similar drivers in wing discs can be easily performed to this end. A western blot probed for total protein and poly-ubiquitin of deltaKASH mutant animals and upon muscle knockdown must be performed to support the assumption that ubiquitinated proteins accumulate in mutant tissues, i.e., that proteostasis is defective. General accumulation of ubiquitinated proteins should appear as a smear by Western Blot.

Extending this analysis to other tissues would be of interest, but is beyond the scope of this paper.

Fig 3 Most Ub positive aggregates colocalize with p62/ref(2)P, known to tag ubiquitinated proteins for autophagic capture and degradation. In agreement with this logic, many Ub puncta are also positive for Atg8a by immunostaining. The authors note that the larger aggregates stain relatively less well for Atg8a whereas it is labeled by the GFP-Atg8 transgene. The authors may want to consider that one possibility for this discrepancy may be that GFP-Atg8a is incorporated as the aggregates form, while antibody staining will stain more easily at the surface. This has been previously observed in several studies.

This is a very plausible hypothesis and we are grateful to this reviewer for the suggestion.

Section: MSP300 reduction led to impaired autophagic flux and to the accumulation of undegraded cargo.

The authors utilize double-tagged GFP-mCherry-Atg8a to assess a defect in autophagic flux. It is true that an increase in double-labeled structures can be indicative of reduced autophagic flux. As this is the central finding of the paper, this hypothesis should be supported by a more direct measure of autophagic flux. One option is to measure autophagic flux by quantifying the amount of tagged Atg8a reaching the lysosome by Western Blot. When GFP or mCherry-tagged Atg8a reaches the lysosome, GFP/mCherry is freed from Atg8a by lysosomal digestion. As both GFP and mCherry are moderately resistant to lysosomal digestion, it can easily be assessed by Western Blot analysis and distinguished from tagged Atg8a due to a shift in size. The amount of mCherry or GFP "alone" is considered a measure of autophagic flux and represents a readily available way to measure autophagy flux in vivo using the tools and experimental regime in the paper.

We used the retention of GFP fluorescence in the Atg8a-tagged transgene as our readout for failure of cargo to reach the lysosome. This readout is a standard in the field.

Figure 5
How was particle volume calculated?

This was presented in the Methods section.

The accumulation of large GFP and Cherry-Atg8a structures that colocalize with polyUb suggests a defective autophagic flux. It would be highly informative to provide EM images, preferably by CLEM or immuno-EM (using anti GFP, Cherry or PolyUb) to diagnose what these structures are. Are the large accumulations of autophagy vesicles, protein aggregates, or some other structures related to muscle formation? This would help identify at what step in autophagic flux MSP300 plays a role.

Probably not muscle formation, as the Drosophila grow about 200 fold (by mass) from the time of muscle formation during embryonic development to the third instar stage. Imaging by EM or CLEM is beyond the scope of this paper.

© 2024. Published by The Company of Biologists under the terms of the Creative Commons Attribution License (https://creativecommons.org/licenses/by/4.0/).

To assess whether MSP300 indeed is required for autophagy flux, the authors should resort to the nutrient-responsive tissue, the fat body. Using tagged GFP-mCherry-Atg8a, the authors can easily perform controlled pulse chase experiments and resolve whether MSP300 is required for autophagy flux with time resolution within a few hours by confocal microscopy. This experiment would have the benefit of extending findings to a non-muscle tissue to exclude functions related to myoblast fusion, for instance. Please consult PMID: 24554766 for instance on how autophagy lysosome fusion defect would manifest phenotypically and how to perform pulse chase assays.

Our interest in studying MSP300 and autophagy relates to possible role in myopathies such as Emery-Dreifuss muscular dystrophy and related neurodegenerative disorders, which is what our NIH grants funded. Therefore, the Drosophila muscle is the appropriate tissue for our studies. The experiments proposed here by this reviewer should be performed by investigators with interests in nutrient responsive tissues.

Minor: Stick to one spelling. PolyUb or PolyUB

Done.

Fig 6 Increased 4EBP/Thor expression indicative of reduced PI3K-TORC1 growth signaling. This would in principle increase autophagy flux and reduce growth consistent with the thinner fibers upon MSP300 but is pursued further as the manuscript now stands.

In addition to decreased PI3K, Foxo is activated by the stress kinase JNK. We are very interest in pursuing mechanistically how Foxo is activated in mutant muscle, but such studies are beyond the scope of this manuscript.

Fig 7. Colocalization experiment of an ER marker and EGFP-tagged MSP300 reveal ER localization, which is interesting, but a bit tangential to the main findings of the paper without further experiments in the direction to show that this location is where autophagy is initiated for instance. Colocalization experiments between Cherry-Atg8a and EGFP-MSP300 upon a starvation regime would be a starting point for such an avenue of experimentation. Some obvious controls are lacking, such as expression of either marker alone to see if they affect each other.

We have imaged ER many times and we find no effect of the MSP300 GFP trap on ER structure. We don't agree that the colocalization study is tangential - the similarity in phenotype between MSP300 mutants and atlastin mutants, in which ER fusion is compromised, led us to ask if MSP300 and atlastin could affect autophagy in the same pathway. Our colocalization studies support this possibility and thereby point the way to common mechanism.

<u>Second decision letter</u>

MS ID#: JOCES/2024/262096

MS TITLE: The Drosophila Nesprin-1 homolog MSP300 is required for muscle autophagy and proteostasis

AUTHORS: Kevin van der Graaf, Saurabh Srivastav, Rajkishor Nishad, Michael Stern, and James A McNew

I am happy to tell you that your manuscript has been accepted for publication in the Journal of Cell Science, pending standard ethics checks.

© 2024. Published by The Company of Biologists under the terms of the Creative Commons Attribution License (https://creativecommons.org/licenses/by/4.0/).
